# Ruthenium(II) Polypyridyl Complexes and Metronidazole Derivatives: A Powerful Combination in the Design of Photoresponsive Antibacterial Agents Effective under Hypoxic Conditions

**DOI:** 10.1021/acs.inorgchem.3c00214

**Published:** 2023-05-10

**Authors:** Gina Elena Giacomazzo, Luca Conti, Camilla Fagorzi, Marco Pagliai, Claudia Andreini, Annalisa Guerri, Brunella Perito, Alessio Mengoni, Barbara Valtancoli, Claudia Giorgi

**Affiliations:** † Department of Chemistry “Ugo Schiff”, University of Florence, Via della Lastruccia 3, Sesto Fiorentino, 50019 Firenze, Italy; ‡ Department of Biology, University of Florence, Via Madonna del Piano 6, Sesto Fiorentino, 50019 Firenze, Italy; § Magnetic Resonance Center (CERM), University of Florence, Via Luigi Sacconi 6, Sesto Fiorentino, 50019 Firenze, Italy

## Abstract

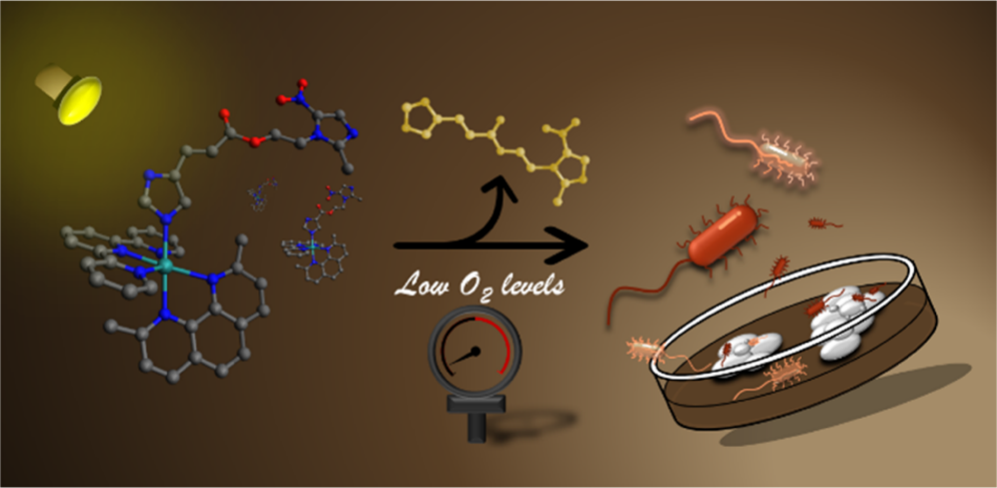

Ruthenium(II) polypyridyl complexes (RPCs) are gaining momentum in photoactivated chemotherapy (PACT), thanks to the possibility of overcoming the classical reliance on molecular oxygen of photodynamic therapy while preserving the selective drug activation by using light. However, notwithstanding the intriguing perspectives, the translation of such an approach in the development of new antimicrobials has been only barely considered. Herein, MTZH-1 and MTZH-2, two novel analogues of metronidazole (MTZ), a mainstay drug in the treatment of anaerobic bacterial infections, were designed and inserted in the strained ruthenium complexes [Ru(tpy)(dmp)(MTZ-1)]PF_6_ (**Ru2**) and [Ru(tpy)(dmp)(MTZ-2)]PF_6_ (**Ru3**) (tpy = terpyridine, dmp = 2,9-dimethyl-1,10-phenanthroline) (Chart 1). Analogously to the parental compound [Ru(tpy)(dmp)(5NIM)]PF_6_ (**Ru1**) (5-nitroimidazolate), the Ru(II)-imidazolate coordination of MTZ derivatives resulted in promising Ru(II) photocages, capable to easily unleash the bioactive ligands upon light irradiation and increase the antibacterial activity against *Bacillus subtilis*, which was chosen as a model of Gram-positive bacteria. The photoreleased 5-nitroimidazole-based ligands led to remarkable phototoxicities under hypoxic conditions (<1% O_2_), with the lead compound **Ru3** that exhibited the highest potency across the series, being comparable to the one of the clinical drug MTZ. Besides, the chemical architectures of MTZ derivatives made their interaction with NimAunfavorable, being NimA a model of reductases responsible for bacterial resistance against 5-nitroimidazole-based antibiotics, thus hinting at their possible use to combat antimicrobial resistance. This work may therefore provide fundamental knowledge in the design of novel photoresponsive tools to be used in the fight against infectious diseases. For the first time, the effectiveness of the “*photorelease antimicrobial therapy*” under therapeutically relevant hypoxic conditions was demonstrated.

## Abstract 2

Optimally designed metronidazole derivatives can be caged into Ru(II) polypyridyl complexes to realize potent antibacterial agents effective under hypoxia and whose activation can be conveniently controlled by light.

## Introduction

Antimicrobial resistance (AMR) is now a leading cause of death worldwide.^1^ Notwithstanding the attention on this global health threat has increased in recent years, the widespread use of antibiotics has dramatically facilitated the emergence of drug-resistant populations of microorganisms, with the result that many hundreds of thousands of deaths are currently due to common, previously treatable, infections. The fight against AMR can no longer wait, and, alongside a more conscious use of antibiotics, there is an urgent need for the development of effective antimicrobials, which should be based on a new class of compounds, rather than on analogues of known scaffolds.

In this respect, transition metal complexes are promising sources for new antimicrobials as they offer augmented electronic properties and a rich variety of three-dimensional structures if compared to their organic counterparts.^2−4^ According to the literature, a number of complexes of transition metals (including Mn, Cu, Zn, Ru, Rh, Pd, Ag, Ir, Pt, Au, *etc*.) were shown to possess antibacterial properties,^5−11^ with few of them that reached the clinical use.^12−14^ However, many opportunities offered by the application of inorganic chemistry to this field of research remain unexplored.

Recently, the encouraging results obtained in the design of antitumoral agents^15−20^ have renewed the interest in Ru(II)-polypyridyl complexes (RPCs), a versatile class of compounds whose antibacterial potential was first reported over 70 years ago.^21,22^ Their rich chemical–physical repertoire, which includes versatile optical and luminescent properties, capacity to interact with key biological targets, and amenability to synthetic tailoring (just to name a few), has been indeed exploited to develop new classes of antibacterial agents.^23−28^ Of particular relevance is the combination of RPCs with light in the so-called antimicrobial photodynamic therapy (aPDT),^29−32^ a technique that relies on the irradiation of a photosensitizer (RPCs) to promote the generation of highly cytotoxic reactive oxygen species (ROS).^33−36^ Besides ROS sensitization, whose effectiveness against both sensitive and multidrug-resistant bacteria has been reported,^37,38^ the main advantage of aPDT consists in the complete spatiotemporal control over the drug activation, which offers the important chance to overcome overdose and side effect issues normally associated with the systemic administration of antimicrobials. However, the reliance of aPDT on molecular oxygen still threatens its application to hypoxic environments, such as anaerobic infections.^39^ This has led to the birth of photoresponsive RPCs able to exert cytotoxic effects *via* O_2_-independent mechanisms, through, for instance, the photorelease of biologically active compounds. Since these processes usually require the population of ligand dissociative metal-centered (^3^MC) states, whose direct excitation is forbidden, strain-inducing substituents are commonly inserted into Ru(II) scaffolds to lower the energy of ^3^MC states and permit their thermal population.^40,41^ In spite of the attractive perspectives, and in net contrast to photoactivated chemotherapy (PACT), where this strategy is gaining momentum,^42−44^ the translation of such an approach in the research of new antimicrobials has been only sparingly investigated. In fact, very few examples^45−47^ followed the pioneering work by Sadler and co-workers on the use of Ru(II) photocages to control the liberation of the antituberculosis drug isoniazid (INH)^48^ and, importantly, none of them inspected the antibacterial potential under the more therapeutically relevant, hypoxic conditions.

**Chart 1:**
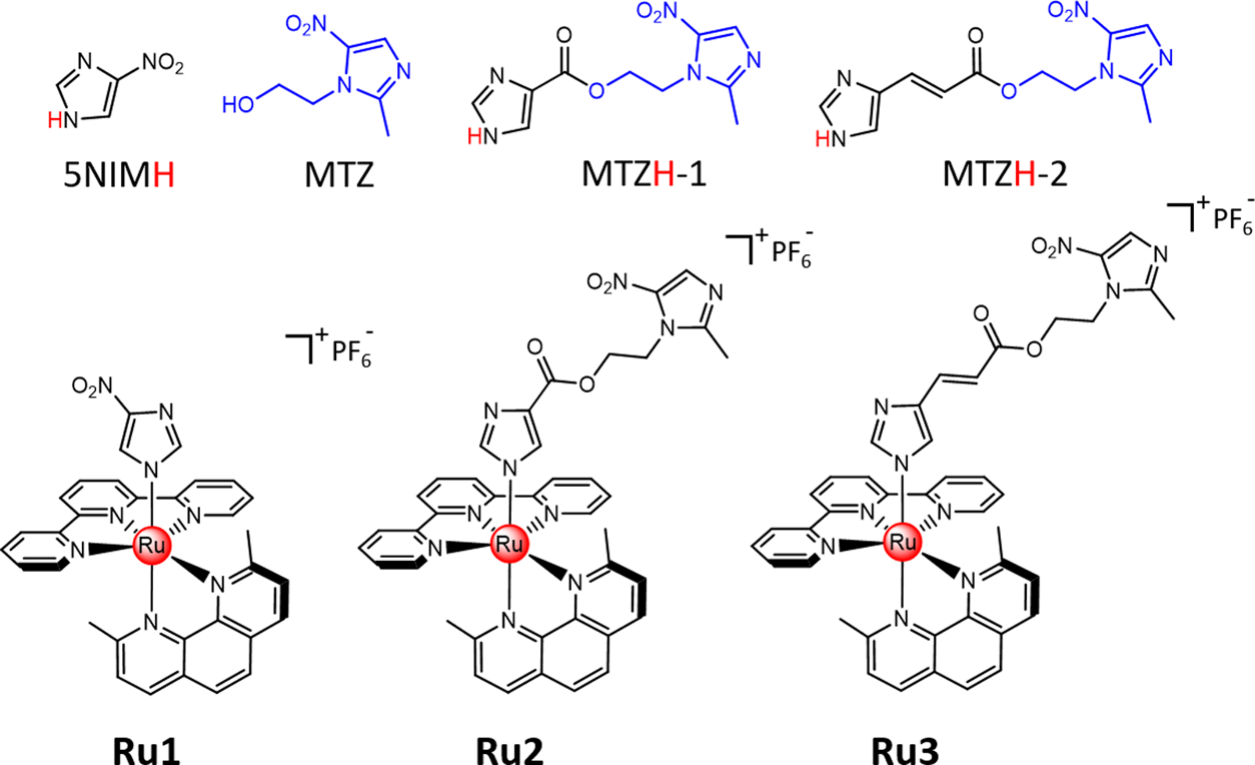
Chemical Structures of Nitroimidazole-Containing Ligands 5NIMH, MTZ, MTZH-1, and MTZH-2 and of the Ruthenium Complexes **Ru1**, **Ru2**, and **Ru3**

With this regard, we recently reported on the combination between RPCs and 5-nitroimidazole (5NIMH), taken as the simplest molecular model of 5-nitroimidazoles,^49^ an intriguing class of broad-spectrum antimicrobial agents, whose peculiar mode of action, based on intracellular bioactivation of the nitro group to toxic radical species, makes them effective even under low-oxygen conditions.^50,51^ We showed that the [Ru(tpy)(dmp)]^2+^ scaffold could be successfully employed to control the photoinduced liberation of 5NIMH from [Ru(tpy)(dmp)(5NIM)]PF_6_ (**Ru1**) (tpy = terpyridine, dmp = 2,9-dimethyl-1,10-phenanthroline) (Chart 1). Indeed, the presence of two bulky methyl groups in the 2 and 9 positions of dmp favored the selective photoejection of the monodentate ligand, enhancing the antibacterial activity against *Bacillus subtilis*, even though only moderate phototoxicities were observed due to the low efficacy of 5NIMH. This prompted us to further explore the use of the [Ru(tpy)(dmp)]^2+^ scaffold to cage more potent nitroimidazole-based antimicrobials.

Herein, MTZH-1 and MTZH-2, two novel derivatives of metronidazole (2-(2-methyl-5-nitro-1*H*-imidazol-1-yl)ethanol), namely, one of the mainstay drugs for the treatment of anaerobic bacterial infections, were synthesized and inserted into the corresponding ruthenium complexes [Ru(tpy)(dmp)(MTZ-1)]PF_6_ (**Ru2**) and [Ru(tpy)(dmp)(MTZ-2)]PF_6_ (**Ru3**) (Chart 1). Besides the characterization of the obtained compounds, their capacity to effectively release the MTZ derivatives upon LED illumination was investigated, whereas their biological activities were inspected against *B. subtilis*, which was chosen as a model of Gram-positive bacteria, *in the dark* and following light exposure, both in normoxic (21% O_2_) and hypoxic (<1% O_2_) conditions. Moreover, the abilities of MTZH-1 and MTZH-2 to interact with NimA, taken as a target protein responsible for metronidazole resistance, were evaluated.

The aim of this study is to demonstrate that RPCs and MTZ derivatives can be combined together to realize promising antibacterial agents, effective under low-oxygen conditions, and whose activation can be conveniently controlled in a spatiotemporal manner by using light. To the best of our knowledge, this study also probes for the first time the effectiveness of the “*photorelease antimicrobial therapy*” approach under hypoxia.

## Experimental Section

### Materials

All materials were of reagent grade and used without further purification. The DMF used for the preparation of the MTZ derivatives was obtained through distillation under a vacuum after using barium oxide as a dehydrating agent.

#### Synthesis of MTZH-1

To a solution of 1*H*-imidazole-5-carboxylic acid (300 mg, 2.68 mmol) in 4 mL of anhydrous DMF, *N*,*N*′-dicycloexylcarbodiimide (DCC, 458 mg, 2.95 mmol) and 4-dimethylaminopyridine (DMAP, 66 mg, 0.54 mmol) were added. After stirring for 20 min at 45 °C, MTZ (493 mg, 2.68 mmol) was added to the reaction mixture, which was left stirring at 45 °C for 2 days. The evaporation under the vacuum of the solvent led to an oil that was dissolved in 200 mL of CH_2_Cl_2_, washed twice with 30 mL of H_2_O, and once with 20 mL of a NaCl-saturated solution. The organic phase was dried over Na_2_SO_4_ and evaporated under reduced pressure. The crude product was purified on silica gel using a DCM/MeOH mixture from 15:1 to 10:1 v/v as an eluent.

Yield 18%. ^1^H NMR (CD_3_OD, 400 MHz): δ 7.95 (s, 1H, H_m_), 7.80 (s, 1H, H_i_) 7.73 (s, 1H, H_j_), 4.78 (t, 2H, *J* = 5.2 Hz, −CH_2_), 4.71 (t, 2H, *J* = 4.8 Hz, −CH_2_), 2.51 (s, 3H, CH_3_) ppm. ^13^C NMR (CD_3_OD, 100 MHz): 151.8, 139.5, 137.9, 131.9, 62.3, 45.8, 13.2 ppm. ESI MS *m*/*z*: calcd for C_10_H_11_N_5_O_4_ [MTZH-1]^+^ (*m*/*z* = 1) 265.08, found 288.08 [MTZH-1 + Na^+^] and 552.83 [2MTZH-1 + Na^+^] (*m*/*z* = 1).

#### Synthesis of MTZH-2

Analogously to the preparation of the metronidazole ester MTZH-1, DCC (370 mg, 2.39 mmol) and DMAP (54 mg, 0.44 mmol) were added to a solution of urocanic acid (300 mg, 2.17 mmol), previously dissolved in 5 mL of anhydrous DMF. The resulting mixture was stirred for 15 min at r.t and, after the addition of MTZ (399 mg, 2.17 mmol), the reaction mixture was left stirring at 45 °C for 4 days. The solvent was then removed in a vacuum, and the resulting oily residue was dissolved in 200 mL of CH_2_Cl_2_, washed twice with 30 mL of H_2_O, and once with 20 mL of a NaCl-saturated solution. The organic phase was dried over Na_2_SO_4_ and evaporated under reduced pressure, affording a crude product that was further purified on silica gel (DCM/MeOH from 20:1 to 10:1 v/v).

Yield 26%. ^1^H NMR ((CD_3_)_2_SO, 400 MHz): δ 8.05 (s, 1H, H_m_), 7.80 (s, 1H, H_i_), 7.56 (s, 1H, H_j_) 7.49 (d, *J* = 15.6 Hz, 1H, −CH), 6.27 (d, *J* = 15.6, 1H, −CH), 4.65 (t, *J* = 4.8 Hz, 2H, −CH_2_), 4.49 (t, *J* = 4.8 Hz, 2H, −CH_2_), 2.48 (s, 3H, −CH_3_). ^13^C NMR ((CD_3_)_2_SO, 100 MHz): 167.2, 152.5, 139.6, 139.0, 134.1, 113.4, 62.9, 46.0, 15.0 ppm. ESI MS *m*/*z*: calcd for C_12_H_13_N_5_O_4_ [MTZH-2]^+^ (*m*/*z* = 1) 291.09, found 314.17 [MTZH-2 + Na^+^] (*m*/*z* = 1).

#### Synthesis of [Ru(tpy)(dmp)(MTZ-1)]PF_6_ (**Ru2**)

MTZH-1 (80 mg, 0.30 mmol) was added to a solution of the ruthenium intermediate [Ru(dmp)(tpy)Cl]PF_6_ (180 mg, 0.25 mmol) in 60 mL of degassed H_2_O-EtOH (50:50% v/v). The reaction mixture was stirred at reflux for 6 h under a N_2_ atmosphere while being protected from light exposure. After cooling at r.t., the addition of a KPF_6_-saturated aqueous solution afforded the precipitation of the ruthenium compound, which was collected by filtration under reduced pressure and washed with water. The crude product was purified by flash chromatography on silica gel (DCM/MeOH 20:1 v/v with 10% of acetone) to obtain complex **Ru2** as a red solid.

Yield 130 mg, 64%.^1^H NMR ((CD_3_)_2_CO, 400 MHz): δ 8.90 (d, *J* = 8.4 Hz, 1H, H_d_ or H_e_), 8.86–8.70 (m, 4H, H_3_, and H_3_), 8.43 (d, *J* = 8.0 Hz, 1H, H_f_ or H_g_), 8.40 (d, *J* = 8.8 Hz, 1H, H_b_ or H_c_), 8.30 (t, *J* = 8.0 Hz, 1H, H_4′_), 8.26–8.04 (m, 6H, H_d_ or H_e_, H_b_ or H_c_, H_6_, H_5_), 7.90 (s, 1H, H_m_), 7.56 (br, 2H, H_4_), 7.44 (d, *J* = 8.0 Hz, 1H, H_f_ or H_g_), 7.24 (s, 1H, H_i_), 6.51 (s, 1H, H_j_), 4.68 (t, *J* = 5.2 Hz, 2H, −CH_2_ MTZ-1), 4.51 (t, *J* = 5.2 Hz, 2H, −CH_2_ MTZ-1), 2.45 (s, 3H, −CH_3_ MTZ-1), 2.32 (s, 3H, CH_3_ dmp), 1.94 (s, 3H, CH_3_ dmp) ppm. ^13^C NMR ((CD_3_)_2_CO, 100 MHz): δ 167.5, 166.9, 159.7, 159.6, 159.0, 154.3, 151.8, 149.1, 148.7, 142.6, 139.2, 137.7, 137.6, 136.2, 133.3, 132.6, 130.1, 129.7, 129.3, 128.3, 127.7, 127.5, 127.1, 126.6, 125.3, 124.4, 63.2, 55.1, 45.2, 25.5, 23.9, 14.1 ppm. HR-ESI MS *m*/*z*: calcd for C_39_H_33_N_10_O_4_Ru [**Ru2** – PF_6_^–^]^+^ (*m*/*z* = 1) 807.17243, found 807.17124 [**Ru2** – KPF_6_^–^]^+^ (*m*/*z* = 1) and 404.09067 [**Ru2** + H^+^- PF_6_^–^]^2+^ (*m*/*z* = 2).

#### Synthesis of [Ru(tpy)(dmp)(MTZ-2)]PF_6_ (**Ru3**)

In analogy to the preparation of **Ru2**, MTZH-2 (73 mg, 0.25 mmol) was added to a solution of [Ru(dmp)(tpy)Cl]PF_6_ (150 mg, 0.21 mmol) in 60 mL of degassed H_2_O–EtOH (50:50% v/v). The reaction mixture was stirred at 70 °C for 7 h under a N_2_ atmosphere while being protected from light exposure and then cooled at r.t. The addition of a saturated KPF_6_ aqueous solution afforded the precipitation of the ruthenium complex, which was collected by filtration under reduced pressure and washed with water. The product was purified by flash chromatography on silica gel (DCM/MeOH 15:1 v/v with 10% of acetone) to obtain complex **Ru3** as a red solid.

Yield 112 mg, 62%. ^1^H NMR ((CD_3_)_2_CO, 400 MHz): δ 8.90 (d, *J* = 8.8 Hz, 1H, H_d_ or H_e_), 8.81 (d, *J* = 8.8 Hz, 2H, H_3′_),8.75 (d, *J* = 7.2 Hz, 2H, H_3_), 8.43 (d, *J* = 8.8 Hz, 1H, H_f_ or H_g_), 8.38 (d, *J* = 8.8 Hz, 1H, H_b_ or H_c_), 8.32 (t, *J* = 8.4 Hz, 1H, H_4′_), 8.25–8.03 (m, 6H, H_d_ or H_e_, H_b_ or H_c_, H_4_, H_6_), 7.88 (s, 1H, H_m_), 7.54 (br, 2H, H5), 7.45 (d, *J* = 8.4 Hz, 1H, H_f_ or H_g_), 7.39 (s, 1H, H_i_), 7.23 (d, J = 17.2 Hz, 1H, −CH MTZ-2), 6.73 (s, 1H, H_j_), 6.15 (d, *J* = 16.4 Hz, 1H, −CH MTZ-2), 4.71 (t, *J* = 5.2, 2H, -CH_2_ MTZ-2), 4.52 (t, *J* = 4.8 Hz, 2H, −CH_2_ MTZ-2), 2.56 (s, 3H, −CH_3_ MTZ-2), 2.44 (s, 3H, −CH_3_ dmp), 1.95 (s, 3H, −CH_3_ dmp) ppm. ^13^C ((CD_3_)_2_CO, 100 MHz): δ 167.4, 167.0, 165.7, 159.7, 154.4, 151.8, 149.1, 148.7, 139.2, 137.7, 137.6, 136.4, 129.2, 128.3, 127.6, 127.5, 127.1, 125.3, 124.4, 117.3, 63.0, 45.5, 25.6, 24.0, 14.0 ppm. HR-ESI MS *m*/*z*: calcd for C_41_H_35_N_10_O_4_Ru [**Ru3** – PF_6_^–^]^+^ (*m*/*z* = 1) 833.18808, found 833.18796 [**Ru3** – PF_6_^–^]^+^ (*m*/*z* = 1) and 417.09812 [**Ru3** + H^+^ – PF_6_^–^]^2+^ (*m*/*z* = 2).

### Methods

During the synthesis of ruthenium compounds, low-light conditions were maintained to avoid any photodecomposition issues. The [Ru(dmp)(tpy)Cl]PF_6_ intermediate was prepared according to methods reported in the literature.^52^

Irradiation of ruthenium complexes was performed by employing a low-energy blue light-emitting diode as a light source (LED, λ_max_ = 434 nm, 160 mW). Photolysis experiments were performed in acetonitrile and in an aqueous solution (PBS buffer, pH 7.4) and were followed by ultraviolet–visible (UV–vis) spectroscopy and high-performance liquid chromatography (HPLC) analyses. In the UV–vis measurements, solutions of **Ru2** and **Ru3** (10 μM) in acetonitrile or aqueous media (total volume of 2 mL) were subjected to increasing irradiation times, and the resulting absorption spectra were collected. In the HPLC measurements, solutions of ruthenium complexes ([Ru] = 100 μM) in aqueous media (total volume of 1 mL) were irradiated for increasing time frames and, at each irradiation point, a 10 μL aliquot of the irradiated solution was injected in the HPLC system. Chromatographic conditions were optimized by using as a gradient mixture of H_2_O/CH_3_CN acidified with 0.1% of formic acid as an eluent, as specified in Table S4 of Supporting Information (SI). The quantum yields for the photodissociation of MTZ derivatives from **Ru2** and **Ru3** (Φ_434_ values) in acetonitrile and water were, respectively, determined through UV–vis spectroscopy and HPLC analysis. Indeed, in water, the close proximity between the ^1^MLCT absorption maximum values corresponding to the starting ruthenium compounds and to the aqua-photoproduct [Ru(tpy)(dmp)(H_2_O)]^2+^ (centered at around 480–490 nm),^53^ along with the broader absorption displayed by **Ru2** and **Ru3** in this solvent, made difficult an equally accurate determination of the Φ_434_ values in water by means of UV–vis measurements. Φ_434_ values were determined by the slope of the linear regression of the moles of the reactant plotted as a function of the moles of the absorbed photons, as previously described.^49^ The photon flux of the light source was obtained by the potassium ferrioxalate actinometry procedure according to methods reported in the literature,^54^ and resulted in being 5.76 × 10^–7^ E/s.

The singlet oxygen-sensitizing properties of ruthenium compounds were investigated through UV–vis analysis by using 1,5-dihydroxynaphthalene (DHN) as an indirect reporter for ^1^O_2_ and according to methods reported in the literature. To this aim, solutions of metal complexes ([Ru] = 10 μM) in aqueous media (PBS buffer, pH 7.4) containing DHN at a concentration of 3.3 × 10^–4^ M were irradiated for progressive time frames for a total period of 10 min. Spectra were acquired by using a solution containing only the ruthenium compound as a blank reference at the same pH and concentration of the measuring cuvette.

The determination of inhibitory concentrations was performed as previously reported.^49^ Briefly, overnight grown liquid cultures of the facultative anaerobic bacterium *B. subtilis* 168 were prepared in 10 mL of an LB medium from single colonies freshly grown on LB agar Petri plates. Cultures were incubated at 37 °C in a rotatory shaker at 225 rpm for 24 h and diluted cultures of *B. subtilis* (OD_600_ = 0.05) were dosed with different concentrations of nitroimidazole-based compounds and ruthenium complexes. The activity of each compound was tested under the dark and following light irradiation, both in normoxia (21% O_2_) and under hypoxic conditions (<1% O_2_). In the photoirradiation experiments, cells were exposed to irradiation (LED emitting light at 434 nm, 1.25 mW) for 40 min; under these conditions, no statistical differences in the blank absorbance between the light- and dark-treated groups of cells were observed. The photon flux in each plate resulted in being 4.72 × 10^–9^ E/s, as determined by the ferrioxalate actinometer method.^54^ Cell growth was carried out statically, by incubating microtiter plates at 37 °C, and it was evaluated by registering the OD_600_ values after 24 h for aerobiosis cultures and after 7 days for the anaerobiosis test. Each test was performed in triplicate, using LB with 0.25% glucose and 0.1% potassium nitrate as culture media to sustain the growth of *B. subtilis* both in aerobiotic and anaerobiotic conditions.^55^ In Figure 3, where the observed antibacterial activities displayed by the tested compounds are reported, the OD_600_ values are normalized and indicate the ratio of cell growth compared to the (untreated) control cells. Differences in the growth were statistically evaluated by the one-way analysis of variance (ANOVA) test and the Tukey post-hoc test (Table S5, SI). The cell growth ratios between different conditions are shown in Figure S35 of SI: lower values indicate lower growth in the first condition tested compared to the second one. Heatmaps were drawn in an R environment, with the package *pheatmap* 1.0.12.

Density functional theory calculations have been carried out with the Gaussian 09 suite of programs^56^ at the B3LYP/6-31+G(d) level of theory. The molecular structure of all studied molecules has been optimized with a very tight criterium, and it has been verified that a minimum has been located by computing the vibrational frequencies, which are all real. Mulliken, Lowdin, and natural population analysis have been performed to determine the atomic charges and to provide further insights into the ligand/ruthenium complex interactions.

### Instrumentation

The ^1^H, ^13^C NMR, COSY, and HSQC spectra were collected with a Bruker 400 MHz spectrometer. Electronic absorption spectroscopy was performed by using a PerkinElmer Lambda 6 spectrophotometer in a 1 × 1 cm^2^ quart cuvette. Fourier transform infrared (FTIR) spectra of compounds were obtained between 500 and 4000 cm^–1^ by a Nicolet iS5 Spectrometer equipped with an iD7 ATR accessory (Thermo Fischer Scientific Inc). HPLC analysis was performed on a Water Alliance 2690 HPLC equipped with a Waters 2487 Abs UV–vis detector set at 270 nm and a KROMASIL 100 Å C–18 150 × 4.6 column. In the determination of inhibitory concentration, the OD 600 values were registered by using an Infinite Pro 200 plate reader (Tecan, Switzerland); the experiments performed under anaerobic conditions were conducted in an anaerobiosis jar (Oxoid jar with Anaerogen 2.5 L), with the system described in paragraph 4 of SI.

## Results and Discussion

### Synthesis and Characterization of Ruthenium Complexes

**Scheme 1:**
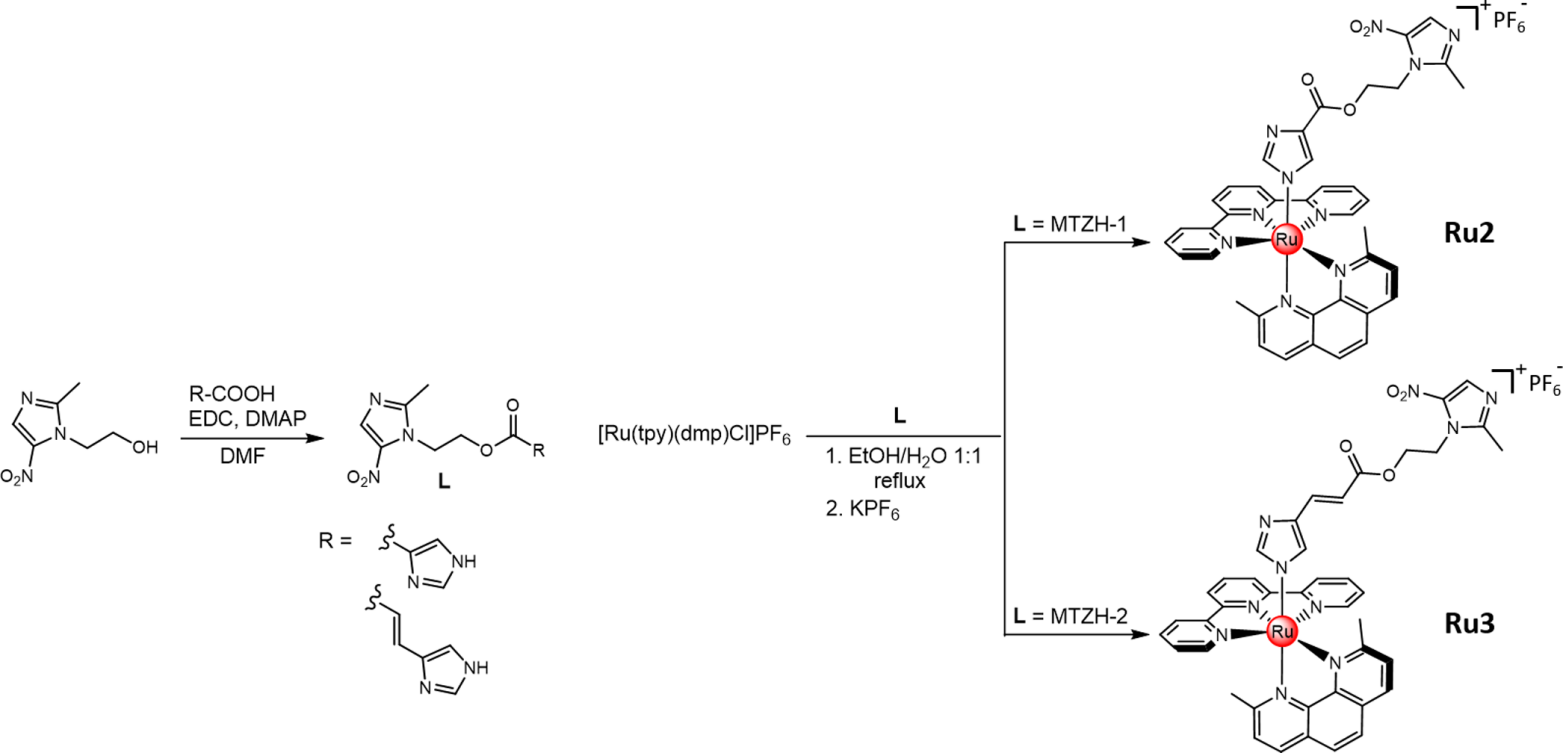
Synthetic Routes Followed for the Synthesis of the Ester Derivatives of Metronidazole MTZH-1 and MTZH-2 and Their Corresponding Ruthenium Complexes **Ru2** and **Ru3**

Metronidazole stands for a reference drug for the treatment of anaerobic infections. However, several anaerobic bacteria and protozoa were shown to develop resistance to MTZ, making it important to research suitable alternatives.^57^ Herein, we designed two analogues of MTZ, in which the clinical drug was coupled with 1*H*-imidazole-5-carboxylic acid (MTZH-1) or urocanic acid (MTZH-2), two imidazole-containing ligands of biological interest and whose imidazole moieties can be exploited as linking units to the Ru(II) centers (*vide infra*). The synthetic routes followed for the synthesis of the metronidazole derivatives MTZH-1 and MTZH-2, as well as for their corresponding ruthenium complexes **Ru2** and **Ru3**, are reported in Scheme 1.

As shown, the MTZ derivatives were synthesized *via* Steglich esterification: MTZ was allowed to react with 1*H*-imidazole-5-carboxylic acid (MTZH-1) or urocanic acid (MTZH-2) in the presence of *N*,*N*′-dicycloexylcarbodiimide (EDC) as a coupling reagent and 4-dimethylaminopyridine (DMAP) as a catalyst. This led to the formation of MTZH-1 and MTZH-2, which were obtained, after purification on silica gel, in 18 and 26% yields, respectively. Their insertion into ruthenium complexes **Ru2** and **Ru3** was accomplished *via* stepwise ligand addition (Scheme 1), in analogy to the synthetic process previously reported for **Ru1**.^49^ Briefly, following the preparation of [Ru(dmp)(tpy)Cl]PF_6_, performed according to the literature,^52,58^ this intermediate was allowed to react with MTZH-1 and MTZH-2 in a hot ethanol–water mixture for 6–7 h under an inert atmosphere, affording the replacement of the chloro ligand with the imidazole rings. Then, the addition of aqueous KPF_6_ led to the precipitation of the hexafluorophosphate salts **Ru2** and **Ru3**, which were obtained, after purification by flash chromatography, in 64 and 62% yields, respectively. The identity of compounds was confirmed through ^1^H, ^13^C, COSY, and HSQC NMR spectroscopy, high-resolution mass spectrometry (HRMS), electron absorption (UV–vis), and attenuated total reflectance (ATR)-FTIR analysis (see SI, Figures S1–S27).

The crystal structure of MTZH-2 was also determined through X-ray single-crystal analysis, as reported in the SI, Figure S12. As shown, the asymmetric unit (a.u.) of MTZH-2 contains two distinct molecules that form intermolecular “*nitrogen*” hydrogen bonds, involving the NH group and the acceptor aza-nitrogen atom, respectively, of their imidazole and nitroimidazole moieties. The occurrence of intramolecular interactions between the −NO_2_ group and the nearest aliphatic H atom, which confer to the molecule a bent conformation, can be also highlighted. Quite interestingly, the whole crystal displayed a singular “*wall–hole*” disposition, where the wall was identified by several molecules of ligands, stabilized by intermolecular bonds and stacking interactions between their imidazole/nitroimidazole rings; these molecules surrounded the resulting channel (hole), which was, in turn, occupied by the solvent molecules identified in the crystal (Figure S12b).

Useful insights into the mode of coordination of MTZ derivatives into ruthenium complexes **Ru2** and **Ru3** came from their HR-ESI MS spectra, which were characterized by the presence of the isotopic patterns relative to the mono positively charged species [Ru(tpy)(dmp)(MTZ-1)]^+^ and [Ru(tpy)(dmp)(MTZ-2)]^+^, respectively, centered at 807.17124 and 833.18795 (*m*/*z* = 1) (Figures S17, S18, S24 and S25 of SI). These data indicated the preferential Ru(II) coordination of MTZH-1 and MTZH-2 by the deprotonated amine nitrogen atom of the imidazolate MTZ-1 and MTZ-2 forms, rather than through their neutral aza-nitrogen atoms (Chart 1), in strict analogy to what was previously found for **Ru1**.^49^ Density functional theory (DFT) calculations performed on MTZ derivatives further corroborated this coordination mode, as denoted by the higher atomic charges determined on the amine nitrogens of the imidazole rings relative to those on the aza-nitrogens gathered on the nitroimidazole moieties of ligands (Table S3). Therefore, in contrast to the “*classical*” Ru(II) coordination by neutral imidazole-based ligands reported for analogue RPCs,^59−62^ these findings suggest that the variable protonation state of the imidazole units,^63^ alongside with metal coordination, would afford Ru(II)-imidazolate species, without the need of strong bases as generally required to preliminary obtain the imidazolate forms of ligands.^64^ Such an unusual coordination mode can be of use to modulate the properties of the resulting metal complexes as photocages, as stronger Ru–N^–^ bonds would ensure higher thermal stabilities while maintaining sufficient ligand photoejection quantum yields (Φ_PS_) (*vide infra*).

**Figure 1:**
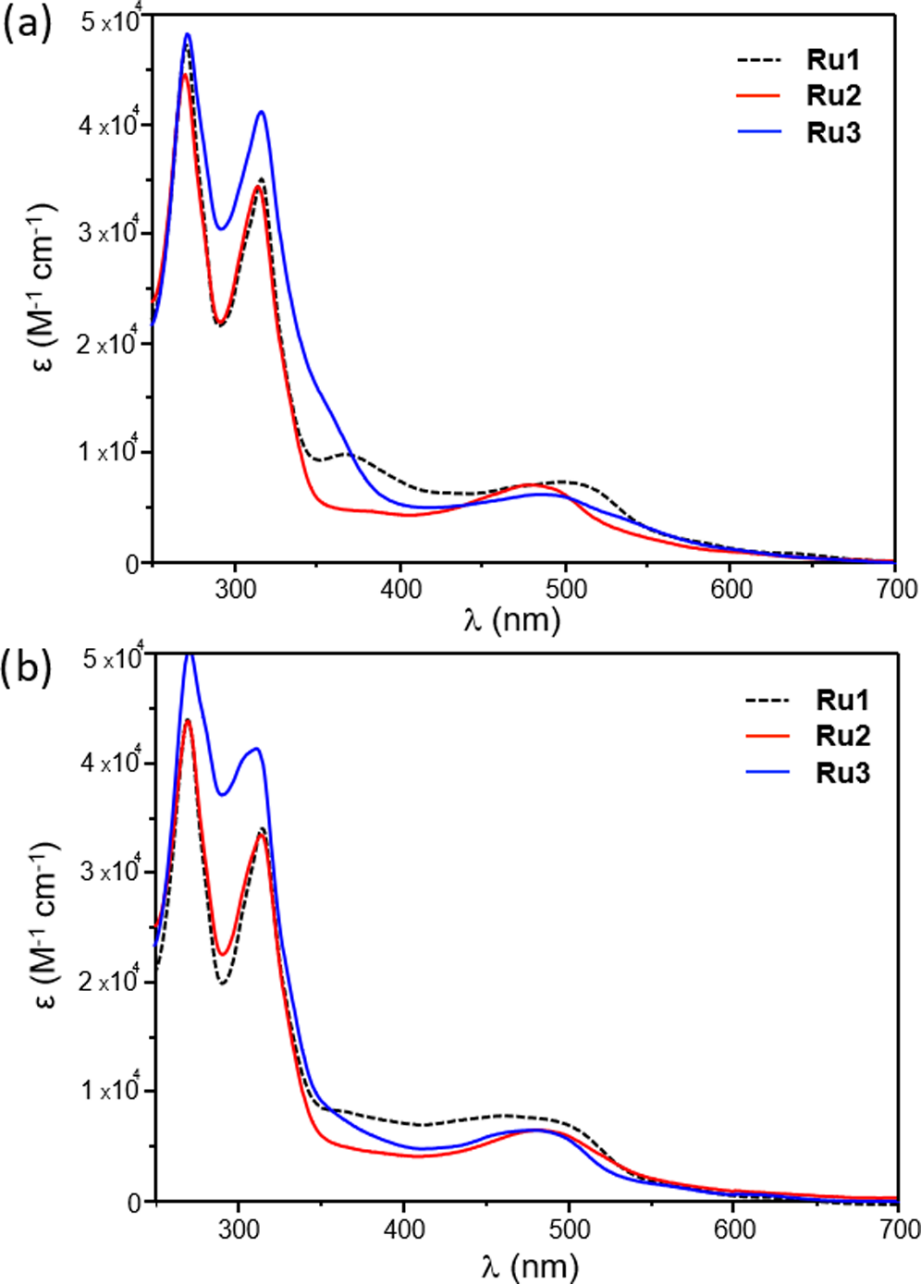
Electronic absorption spectra of **Ru1**, **Ru2**, and **Ru3** in acetonitrile (a) and in a PBS buffer solution, pH 7.4 (b).

The absorption properties of ruthenium complexes were also considered. Figure 1 reports the electronic absorption spectra of **Ru2** and **Ru3** in acetonitrile and in an aqueous solution (PBS buffer, pH 7.4), along with the one of **Ru1** for comparison. As generally found for RPCs,^59^ these compounds display the typical ^1^ππ* transitions relative to tpy and dmp ligands in the UV region along with a broad, unresolved metal-to-ligand Ru(dπ) → tpy/dmp(π*) charge transfer (^1^MLCT) band in the visible range. In acetonitrile, the lowest energy absorption bands of **Ru2** and **Ru3** are slightly blue-shifted compared to **Ru1**, with maximum values centered at 482 nm (ε = 7124 M^–1^ cm^–1^), 492 nm (ε = 6167 M^–1^ cm^–1^), and 500 nm (ε = 7344 M^–1^ cm^–1^) for **Ru2**, **Ru3**, and **Ru1**, respectively (also see Table 1). Smaller variations between the wavelengths corresponding to the maxima of ^1^MLCT transitions were displayed in aqueous media. A residual absorption tail within the 550–650 nm range of the spectra can be also evidenced for all of the complexes, thus providing a promising feature for their activation by using low-energy light, with enhanced depth penetration into tissues. On the other side, these complexes emerged to be weakly emissive, likely due to nonradiative decay pathways promoted by the competitive population of ^3^MC states.^65^

**Table 1:** Absorption Maxima and Photoinduced Ligand Substitution Quantum Yields (Φ_434_) for **Ru1**, **Ru2**, and **Ru3**

complex	λ_max_/nm (ε/M^–1^ cm^–1^) in CH_3_CN	λ_max_/nm (ε/M^–1^ cm^–1^) in H_2_O	Φ _434_ in CH_3_CN^a^	Φ _434_ in H_2_O^b^
**Ru1**	498 (7349)	462/490 (7831/7438)	0.0039 (3)^c^	0.0011 (3)^c^
**Ru2**	480 (7006)	485 (6475)	0.0079 (9)	0.00027 (2)
**Ru3**	488 (6200)	480 (6442)	0.0059 (4)	0.0015 (2)

a Determined through UV–vis measurements.

b obtained by HPLC analysis.

c Ref (49).

### Photoreactivity of Ruthenium Complexes

**Figure 2:**
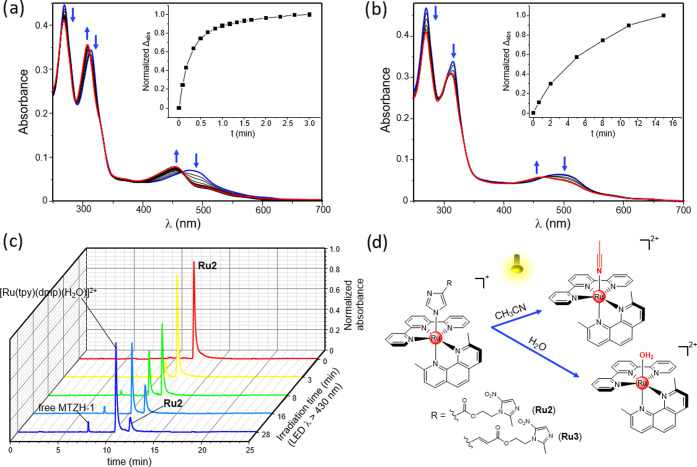
UV–vis absorption spectra of **Ru2** in acetonitrile (a) and in water at neutral pH (b) registered at different irradiation times. Photolysis of **Ru2** in an aqueous solution monitored by means of HPLC analysis (c). Proposed ligand photoejection processes underwent by **Ru2** and **Ru3** (d).

The ability of **Ru2** and **Ru3** to release the MTZ derivatives upon light irradiation was explored in acetonitrile and in an aqueous solution (PBS buffer, pH 7.4) by coupling UV–vis and HPLC analyses. The obtained results for **Ru2** and **Ru3** are, respectively, reported in Figures 2 and S30 and S31 of SI.

Contrary to dark conditions, in which, analogously to **Ru1**, these systems displayed remarkable stability (Figures S28 and S29, SI), visible-light exposure (LED emitting at 434 nm, 160 mW) determined clear changes in the electronic absorption spectra of both compounds, indicating the occurrence of photoejection processes. For instance, as shown in Figure 2a for acetonitrile solutions of **Ru2**, light exposure promoted a sharp blue shift in the ^1^MLCT absorption maximum of the metal complex, from 480 to 454 nm, indicating the substitution of MTZH-1 with a solvent molecule to give [Ru(tpy)(dmp)(CH_3_CN)]^2+^.^66−68^ Photolysis of **Ru2** also took place in aqueous media, albeit on a larger time scale (Figure 2b), thus providing a key prerequisite for biological applications. Importantly, the presence in the UV–vis titrations of multiple isosbestic points indicated the direct conversion to single photoproducts. Parallel HPLC experiments provided further evidence of the selectivity of the photoejection processes. Indeed, as shown in Figure 2c, where the chromatograms of aqueous solutions of **Ru2** exposed to increasing irradiation times are reported, light irradiation promoted the progressive decrease of the peak associated with the starting metal complex (retention time *t*_r_ of 11.77 min), accompanied by the appearance of two new peaks at 7.42 and 10.35 min, which can be, respectively, attributable to the liberated MTZH-1 and the resulting Ru(II) aquocomplex. The lack of evidence of free tpy or dmp confirmed the selective photorelease of the monodentate imidazole ligand. Analogue results were also obtained for **Ru3**, as shown by photolysis experiments reported in Figures S30 and S31 of SI.

The quantum yields for the ligand photodissociation from **Ru2** and **Ru3** (Φ_434_ values) were determined through the UV–vis and HPLC analyses as previously described and following the determination of the LED photon flux by the potassium ferrioxalate actinometry procedure.^49,54^ The obtained results are summarized, along with the ones previously determined for **Ru1**, as shown in Table 1. As shown, the light-induced detachment of nitroimidazole-containing ligands in water occurred with Φ_434_ values ranging from 0.00027(2) to 0.0015(2), with **Ru3** being the most efficient. Higher values, of 0.0079 (9) (**Ru2**) and 0.0059 (4) (**Ru3**), were found in acetonitrile, indicating a higher efficiency of photolysis in organic media. Overall, it can be noted that the results determined for **Ru1**, **Ru2**, and **Ru3** turned out to be at least one order of magnitude lower if compared to the parental compound [Ru(tpy)(dmp)(py)]^2+^ (py = pyridine), for whom, for example, the photorelease of a py unit in acetonitrile was reported to take place with a quantum yield of 0.058.^53^ On the other hand, the lower photoreactivities of **Ru1**, **Ru2**, and **Ru3** were counterbalanced by remarkable stabilities displayed in dark conditions (Figures S28 and 29, SI). Therefore, apart from the different stereoelectronic features of ruthenium complexes, we can speculate that the peculiar Ru(II)–N^–^ coordination of nitroimidazole-containing ligands may affect both their thermal stability and photoreactivity, tuning the properties of the resulting complexes as photocages. Accordingly, this class of monodentate ligands would represent a suitable alternative to more extensively explored leaving groups, such as pyridines,^69^ pyrazines,^54^ amines,^70^ thioethers,^71^ and nitriles,^72^ in an effort to answer the increasing demand for the development of new Ru(II)-based photocages, with sufficient ligand photoejection quantum yields without a concomitant loss in thermal stability.^53^

Lastly, since various pathways are, in principle, made accessible by irradiation, the capacity of ruthenium complexes to sensitize the production of singlet oxygen (^1^O_2_) was also evaluated. To this aim, aqueous solutions of metal compounds, in the presence of 1,5-dihydroxynaphtalene (DHN) as an indirect probe for ^1^O_2_, were exposed to progressive irradiation times, and the resulting UV–vis spectra were collected. As shown in Figure S32 (SI), no evidence of the formation of Juglone, namely the photo-oxidation product of DHN, was observed during the overall time frame investigated. This confirms, in analogy to **Ru1**, the scarce sensitizing properties of **Ru2** and **Ru3** and further supports the population of dissociative ^3^MC states, which can be harnessed to exert cytotoxic effects in low-oxygen conditions.^73^

### *In Vitro* Antibacterial Activity and Interaction with NimA

Preliminary to the evaluation of the antibacterial properties of Ru(II) compounds, their capacity to be effectively internalized by *B. subtilis* strain 168, which was selected as a model of Gram-positive bacteria, was investigated through inductively coupled plasma atomic emission spectroscopy (ICP-AES), as described in the SI (paragraph 3). As shown in Figure S33, all three complexes were found to be successfully internalized by bacterial cells, with the cell uptake following the order **Ru3** > **Ru1** > **Ru2**, thus suggesting a subtle influence of the different nitroimidazole derivatives on the permeation abilities of their resulting Ru(II) complexes.

**Figure 3:**
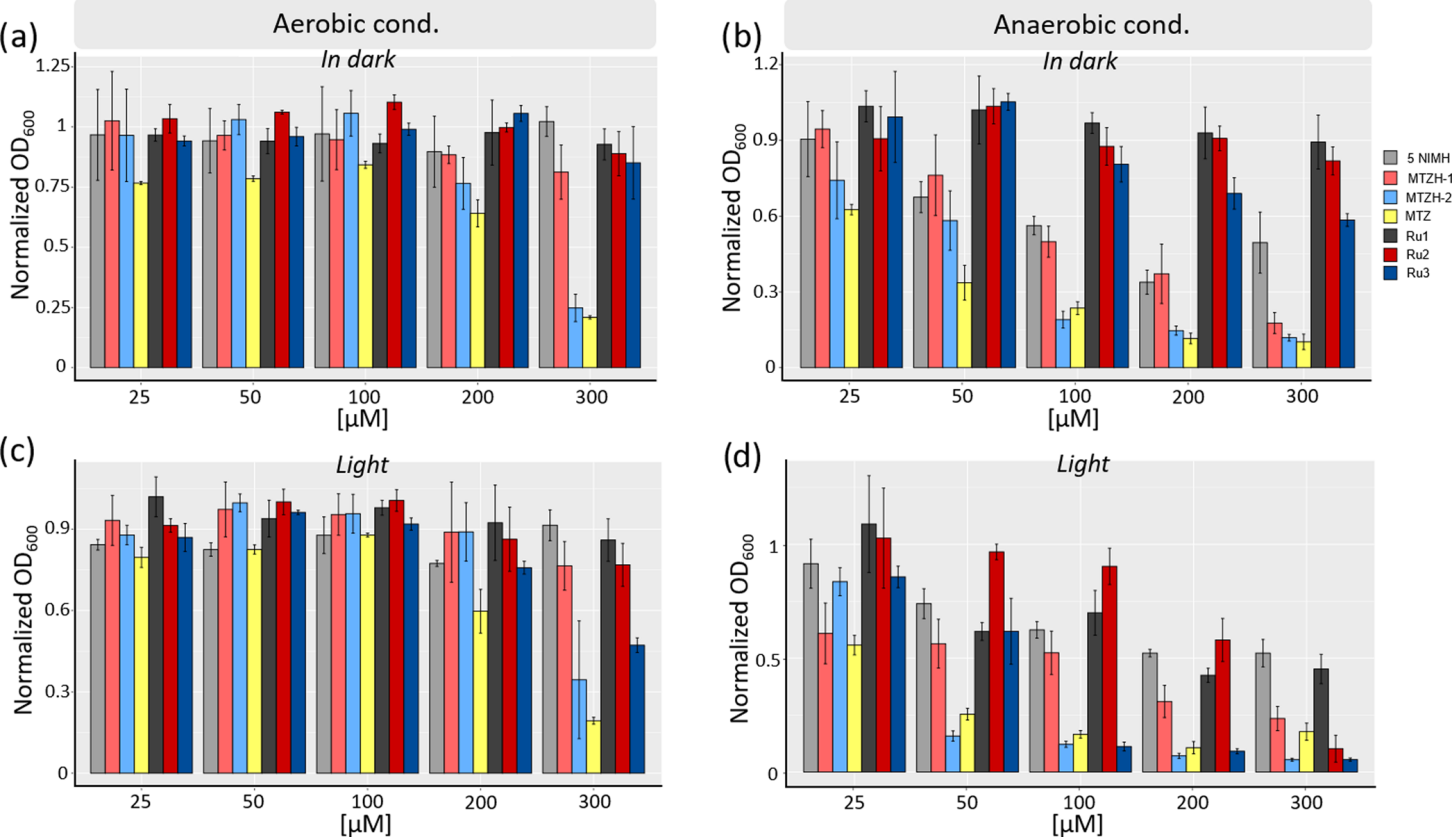
Antibacterial effect of ruthenium photocages **Ru1**, **Ru2**, and **Ru3**, their corresponding imidazole-based ligands 5NIMH, MTZH-1, and MTZH-2, and MTZ evaluated in the dark and following light activation under both aerobic (a, c) and anaerobic (b, d) conditions. Ratios of the normalized OD_600_ values are reported with respect to the blank control (*Y*-axis) as a function of different drug concentrations ([μM], *X*-axis).

We then inspected the antibacterial activities of **Ru1**, **Ru2**, and **Ru3**, along with the ones of their corresponding nitroimidazole-based ligands 5NIMH, MTZH-1, and MTZH-2; the effect of MTZ was also analyzed for comparison. Experiments were performed in the *dark* and following blue-light irradiation (LED emitting at 434 nm, *t* = 40 min) both in normoxia (21% O_2_) and in hypoxia (<1% O_2_), by using the experimental setup described in the SI (paragraph 4). The obtained results, expressed as normalized OD_600_ values indicating the ratio of cell growth compared to the control (untreated) cells, are reported in Figure 3.

As shown in Figure 3a, *in* the *dark* and under aerobic conditions, all of the tested compounds generally displayed negligible toxicity, except for MTZ and MTZH-2, which led to a remarkable effect starting at relatively high drug doses (>200 μM), with a *ca.* 75% reduction of cell growth at a higher concentration tested (300 μM). Irradiation under these conditions slightly affected the activities of the investigated compounds (Figure 3c). Indeed, only modest phototoxicities were observed for high doses of ruthenium complexes, with **Ru3** being the most effective compound across the series, featuring a *ca*. 40% reduction of cell growth at 300 μM.

A completely different scenario was observed under anaerobiosis. As it can be noted from Figure 3b, in the dark, all of the MTZ-containing compounds exhibited a sharp dose-dependent activity, with MTZH-2 and MTZ that displayed the highest, and comparable, effects. For instance, at a concentration of 100 μM, MTZH-2 and MTZ induced a reduction of cell growth around 75%, whereas a lower, though remarkable, effect (*ca*. 50%) was found for MTZH-1 at the same dose. Interestingly enough, even the less potent 5NIMH was found to induce a net dose-dependent effect under these conditions, in contrast to the scarce efficacy displayed in the aerobic tests of the present and previous reports.^49,74^ The superior effectiveness of nitroimidazole-based compounds observed under anaerobic compared to aerobic conditions can be rationalized by considering the generally accepted mode of action of nitroimidazoles.^50^ Indeed, this class of antimicrobials typically relies on the preliminary bioactivation of the nitro group, which occurs through its intracellular reduction to a radical anion to result in harmful ROS species, capable of interacting with a variety of cellular targets. However, high oxygen levels cause the rapid reoxidation of the radical anion, resulting in a futile cycle that impairs the bactericidal potential under aerobic conditions.

From Figure 3b, it also emerges that the antibacterial effects of “*Ru-free*” nitroimidazole-based ligands are effectively masked by their inclusion into ruthenium complexes, as indicated by the good tolerability demonstrated by cells toward **Ru1**, **Ru2**, and **Ru3** in the dark. Among these complexes, it can be highlighted the highest toxicity of **Ru3**, which, however, did not exceed a *ca*. 48% reduction of cell growth at the maximum dose tested.

Instead, the bioactivities of these ligands were partially or almost fully recovered upon irradiation under anaerobiosis, pointing at a different behavior to what is commonly observed in PACT, where the light-mediated effects are severely jeopardized when switching from aerobic to anaerobic conditions.^73,75^ In fact, as shown in Figure 3d, the three ruthenium complexes displayed a sharp dose-dependent effect, with **Ru3** being the lead compound across the series. For instance, light irradiation of this complex at a concentration of 100 μM unleashed a *ca.* 9-fold enhancement of activity compared to dark conditions and induced, yet for drug doses above 50 μM, a comparable potency to the one of MTZ. In other words, the adoption of the photocaging strategy to control the liberation of this MTZ derivative would allow reproducing the high effectiveness of MTZ under hypoxic conditions but with the key advantage arising from the spatiotemporal control over the drug activation ensured by the use of light. It can be also noted that the phototoxicity of this complex nicely paralleled the effect of the *Ru-free* MTZH-2 derivative, thus making the liberation of this potent bioactive compound likely to be the cause of the observed photoactivity.

Lower, though remarkable, phototoxicities were induced by **Ru1** and **Ru2**, with **Ru2** that was the least effective in the 0–200 μM range. Its relatively low efficacy can be tentatively rationalized by considering that, besides the higher antibacterial activity of MTZH-1 relative to 5NIMH, the latter ligand was more efficiently photoreleased in aqueous media than MTZH-1 (Table 1). A rough correlation between the observed antibacterial activities of Ru(II) complexes and their cellular uptake should be also pointed out. This would suggest that an optimal balance between the biological activity of “*caged*” ligands, their capacity to be easily released from Ru(II)-photocages and the permeation abilities of the intact Ru(II) complexes, is crucial for the phototoxicity of the resulting Ru(II)-based antimicrobials.

Furthermore, the following remarks can be highlighted from the cell growth ratios registered between the different conditions tested, reported in Figure S35 of the SI: (i) the efficacy of nitroimidazole-based compounds is clearly enhanced in anaerobic compared to aerobic conditions and (ii) in neither aerobic nor anaerobic conditions, their antibacterial activity is markedly affected by light activation. Instead, irradiation in hypoxia strongly improved the dose-dependent activities of the photolabile complexes **Ru1**, **Ru2**, and **Ru3**, with **Ru3** featuring the highest phototoxicity across the series. Such results were also confirmed by measuring the cell viability after exposure to anaerobic conditions; as shown in Table S5 of SI, the effect of the photoactivated complexes under hypoxia not only reduced cell growth but also cell viability (*i.e.*, photoactivated complexes showed a bactericidal activity), with a complete killing of bacterial cells at 200 μM photoactivated **Ru2** and **Ru3** complexes. We also specify that effects on growth kinetics, and not only on overall growth and viability, can be present. However, the experimental setting of anaerobic conditions strongly limited the possibility of measuring continuous growth kinetic data, which were then not included in the present study.

Lastly, bacterial resistance toward 5-nitroimidazole-based antibiotics is generally believed to be associated with decreased drug uptake and/or altered/deficient reduction efficiency. Concerning the latter point, several classes of resistant bacterial strains have been shown to possess *Nim* genes, which encode for reductase enzymes capable of converting the nitro group of the drug into a nonbactericidal amine and thus preventing the accumulation of the toxic nitro radical. More in detail, a previous study reporting on the crystal structure of NimA from *Deinococcus radiodurans* (drNimA) complexed with MTZ^76^ proposed that the inactivation of this antibiotic would be the result of a two-electron reduction process taking place in the active site of the protein and mediated by the covalent binding of the cofactor pyruvate with His-71. Therefore, being inspired by these findings, we further considered the interaction of MTZ derivatives with the active site of NimA, which was selected as a representative model for this family of reductases.

**Figure 4:**
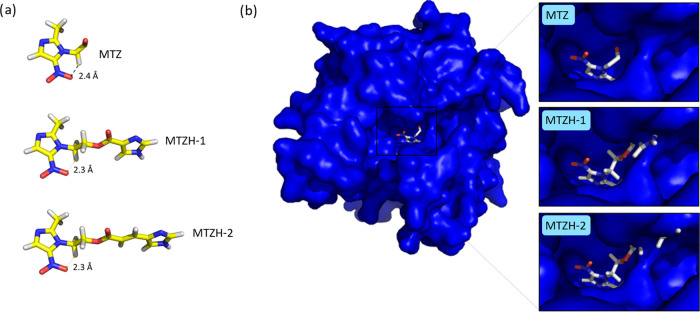
Experimental MTZ structure in the 1W3R PDB file and optimized MTZH-1 and MTZH-2 structures computed through DFT calculations (a) and MTZ, MTZH-1, and MTZH-2 ligands interacting with NimA protein (the surface of the protein is marked in in blue) (b).

To this aim, the molecular structures of MTZ, MTZH-1, and MTZH-2 ligands were optimized with DFT calculations at the B3LYP/6-31+G(d) level of theory. The obtained results, shown in Figure 4a, evidenced that these ligands are characterized by an intramolecular hydrogen bond between the −NO_2_ group of the nitroimidazole residue and the nearest hydrogen atom of a −CH_2_ residue, in good agreement with the X-ray data above discussed for MTZH-2 (also see SI, paragraph 1.2). It can be noted that such a hydrogen bond reduces the mobility of the ring moiety with respect to the substituents, and a similar intramolecular bond can be also observed in the experimental MTZ structure present in the PDB file 1W3R, thus confirming the agreement between experimental and our computed ligands. Then, the ring of each compound was superposed onto MTZ in 1W3R in NimA obtaining the results reported in Figure 4b. As shown, the rigidity imparted by the intramolecular interactions combined with the more extended chains gathered on the ester functions of MTZH-1 and MTZH-2 made these compounds clash with the NimA protein structure, whereas MTZ is nicely accommodated within the active site of the protein. Therefore, these preliminary data suggest that the different chemical structures of the two MTZ derivatives would impact their susceptibility to drug inactivation by NimA and hint at their possible use as potential options for clinicians to manage resistance to nitroimidazole-based antibiotics.

## Conclusions

The net disparity between the countless examples of PACT agents and those studied for the photorelease of bioactive ligands in the design of new antimicrobials prompted us to design two novel derivatives of the antibiotics MTZ, MTZH-1, and MTZH-2, which were inserted in the corresponding strained ruthenium complexes **Ru2** and **Ru3**. The two MTZ derivatives were synthesized by Steglich esterification, by coupling MTZ with different imidazole-containing ligands of biological interest and whose imidazole moieties were exploited as linking units to Ru(II) centers. Analogously to the parental compound **Ru1**, the unusual Ru(II) coordination of MTZ derivatives in their imidazolate forms conferred the resulting Ru(II) complexes promising features as photocages, as denoted by the good stability shown in the *dark* and by the possibility to easily cause the detachment of MTZ-based ligands from ruthenium scaffolds upon exposure to visible light. The biological potential of metal-free ligands and metal complexes was tested on *B. subtilis*, which was chosen as a model of Gram-positive bacteria, and experiments were conducted both under normoxic (21% O_2_) and hypoxic (<1% O_2_) conditions. Our results highlighted two main findings: (i) the activity of metronidazole-containing ligands was remarkably enhanced by switching from aerobic to anaerobic conditions, as expected in consideration of the peculiar mode of action of the class of nitroimidazole-based antimicrobials. (ii) The insertion of ligands into ruthenium complexes masked their activity when kept in the *dark*, whereas light irradiation under hypoxia provoked a strong dose-dependent activity, with the lead compound **Ru3** that unleashed a comparable effect to one of the MTZ. These findings, therefore, probed that the photocaging strategy can be successfully exploited to control the activity of optimally designed MTZ derivatives, affording remarkable antibacterial activities under low-oxygen conditions upon light activation of the prodrugs.

Lastly, preliminary studies on the interaction of the MTZ derivatives with NimA, chosen as a model of reductases responsible for bacterial resistance against 5-nitroimidazole-based antibiotics, unveiled that the different chemical architectures of these bioactive ligands made their protein sequestration unfavorable if compared to MTZ, thus hinting at their possible use in the treatment of bacteria resistant to nitroimidazole-based antimicrobials.

In conclusion, the results of this work may provide fundamental knowledge for the development of suitable alternatives to be used in the struggle against infectious diseases, effective under hypoxic conditions and whose activation could be selectively controlled by using light. Moreover, for the first time, the effectiveness of the “*photorelease antimicrobial therapy*” approach under hypoxia was demonstrated.

## Supporting Information Available

NMR, MS, ATR-FTIR, UV–vis, and fluorescence spectra of compounds; X-ray and DFT analysis; ICP-AES analysis; materials and methods; and experimental details, including photographs of the experimental setup for the antibacterial tests (PDF)
