# Supplementary material for: Ruthenium(II) Polypyridyl Complexes and Metronidazole Derivatives: A Powerful Combination in the Design of Photoresponsive Antibacterial Agents Effective under Hypoxic Conditions
Source: Inorg Chem. 2023 May 10;62(20):7716–27. doi: 10.1021/acs.inorgchem.3c00214 (PMC10207323; doi:10.1021/acs.inorgchem.3c00214)
Supplement: Supplementary file 1 — ic3c00214_si_001.pdf [file ic3c00214_si_001.pdf]

Supporting Information

Ruthenium(II) polypyridyl complexes and metronidazole derivatives: a powerful combination in the design of photoresponsive antibacterial agents effective under hypoxic conditions

Gina Elena Giacomazzo,[a] *Luca Conti,*[a*] *Camilla Fagorzi,*[b] *Marco Pagliai,*[a] *Claudia Andreini,*[a,c] *Annalisa Guerri,*[a] *Brunella Perito,*[b] *Alessio Mengoni,*[b] *Barbara Valtancoli*[a] *and Claudia Giorgi.*[a*]

[a] Department of Chemistry "*Ugo Schiff*", University of Florence, Via della Lastruccia 3, 50019, Sesto Fiorentino (FI), Italy.

[b] Department of Biology, University of Florence, Via Madonna del Piano 6, 50019, Sesto Fiorentino (FI), Italy.

[c] Magnetic Resonance Center (CERM), University of Florence, Via Luigi Sacconi 6, 50019, Sesto Fiorentino (FI), Italy

Corresponding author's email address: luca.conti@unifi.it, claudia.giorgi@unifi.it

Table of Contents

1. *Characterization of compounds*

    1.1. *Characterization of MTZ-derivatives*

    1.2. *X Ray crystallography*

    1.3. *Characterization of ruthenium compounds*

2. *Photoreactivity of ruthenium complexes*

    2.1. *Stability of ruthenium compounds under dark*

    2.2. *Photoejection of MTZ derivatives*

    2.3. *Singlet oxygen generation*

3. *Cellular internalization by ruthenium compounds*

4. *Determination of the inhibitory concentration*

1. Characterization of compounds

1.1. Characterization of MTZ-derivatives

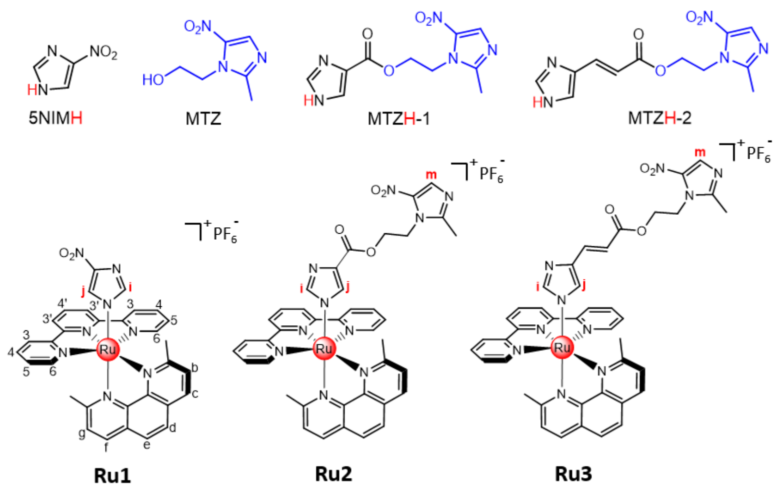

Chart S1. Chemical structures of 5NIMH, MTZ, MTZH-1, MTZH-2 and of the ruthenium complexes **Ru1**-**3**, along with the labelling used for signal attribution in the NMR analysis of ruthenium complexes.

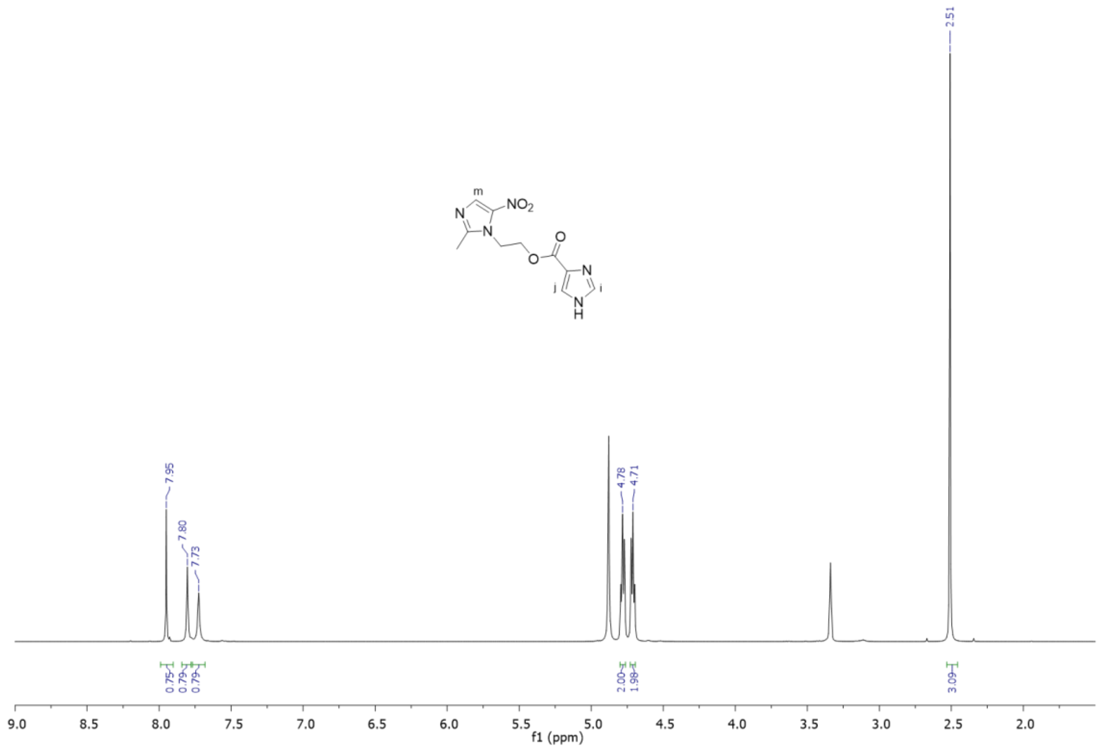

Figure S1. ^{1}H NMR (400 MHz) spectrum of MTZH-1 in CD_3OD.

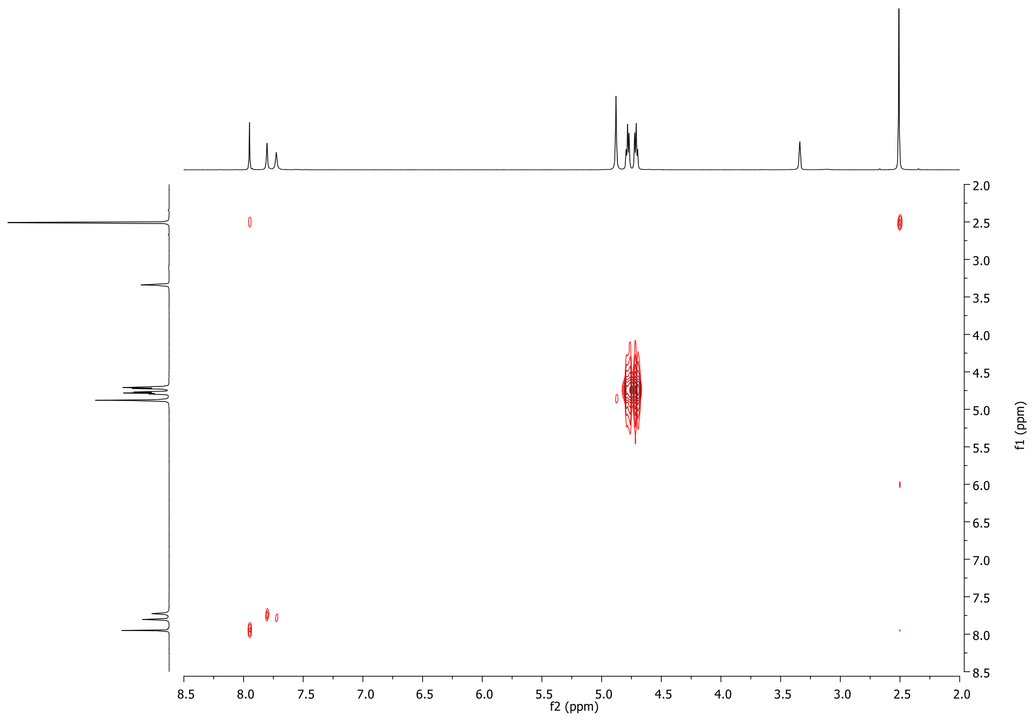

Figure S2. COSY spectrum of MTZH-1 in CD_3OD.

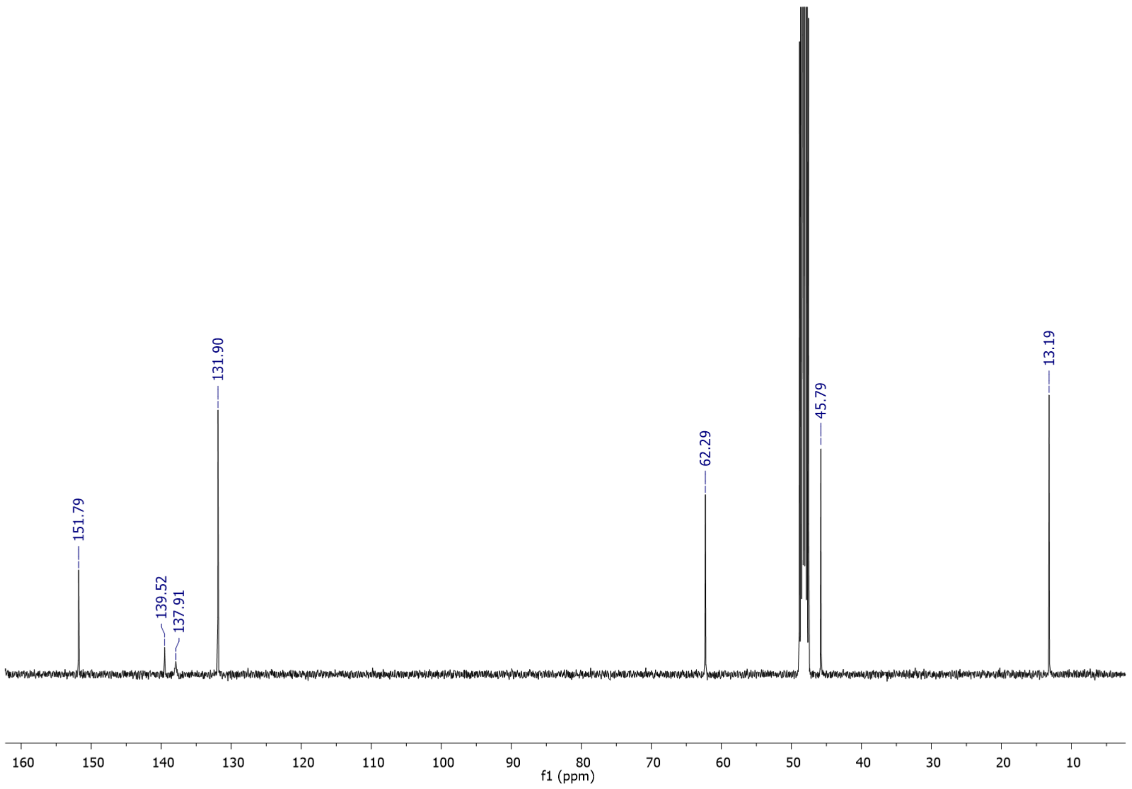

Figure S3. ^{13}C NMR (100 MHz) spectrum of MTZH-1 in CD_3OD.

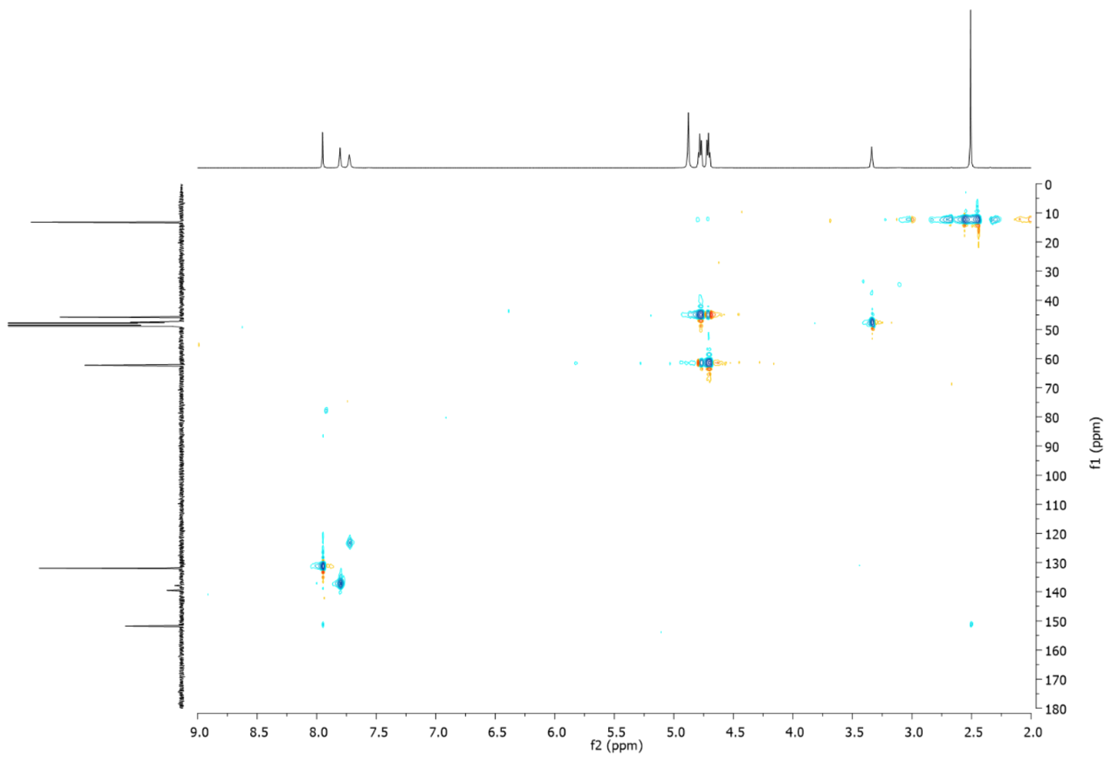

Figure S4. HSQC spectrum of MTZH-1 in CD_3OD.

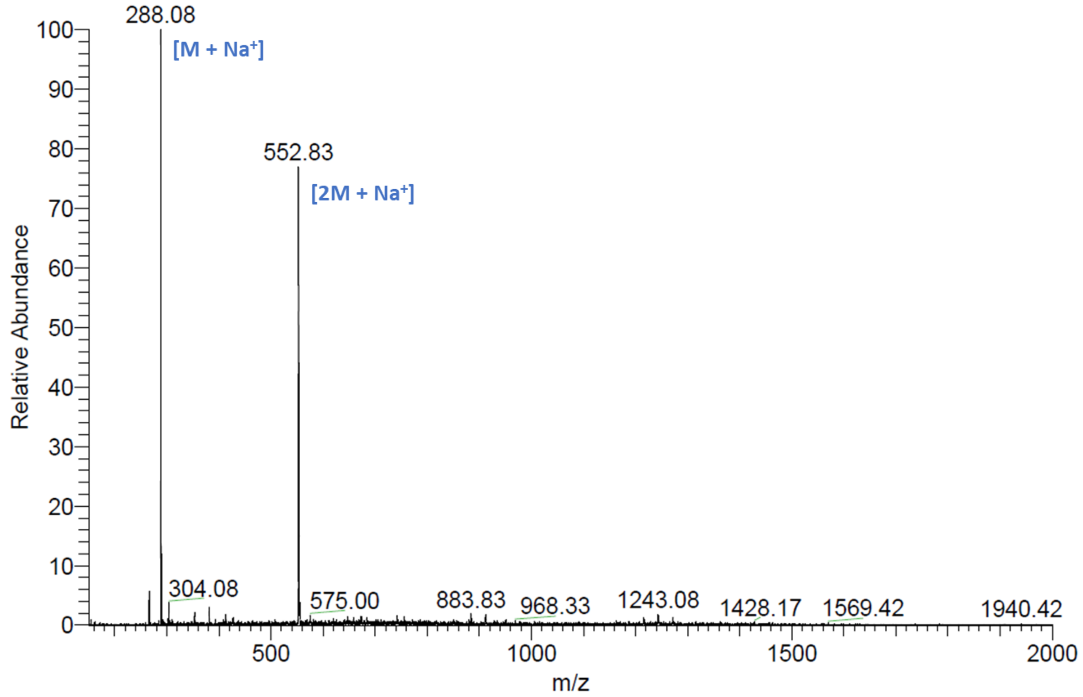

Figure S5. (ESI+)-MS spectrum of MTZH-1 (M) in methanol.

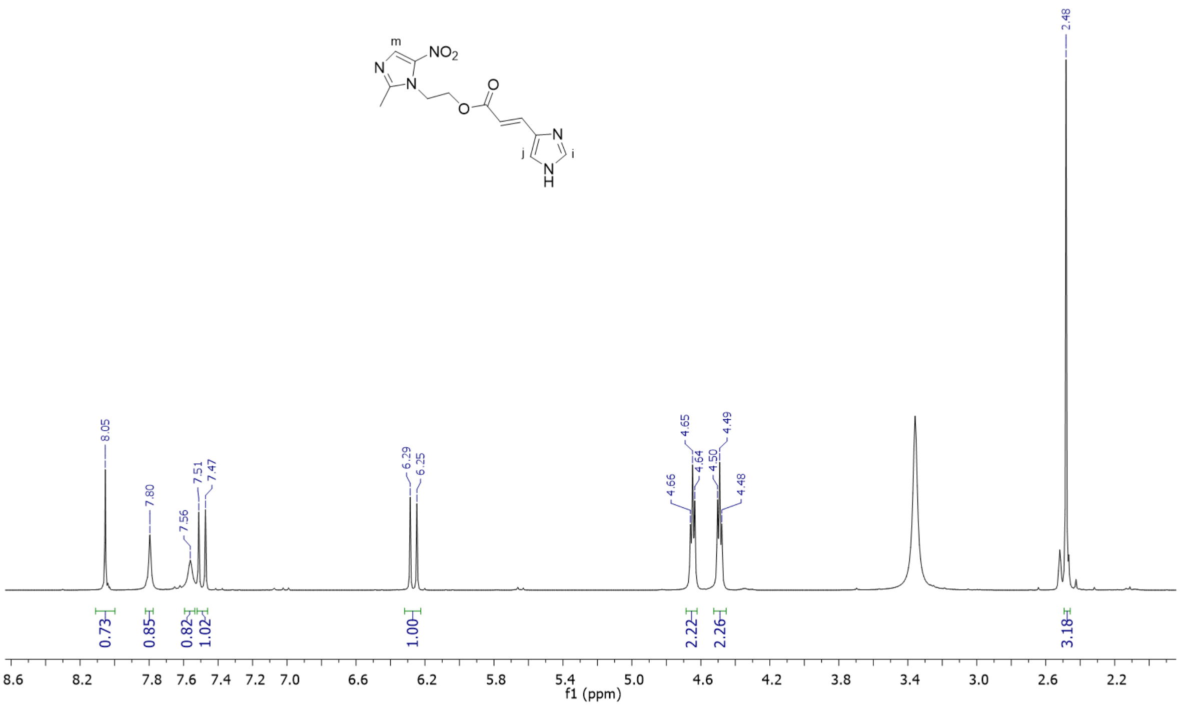

Figure S6. ^{1}H NMR (400 MHz) spectrum of MTZH-2 in $(CD_3)_2SO$.

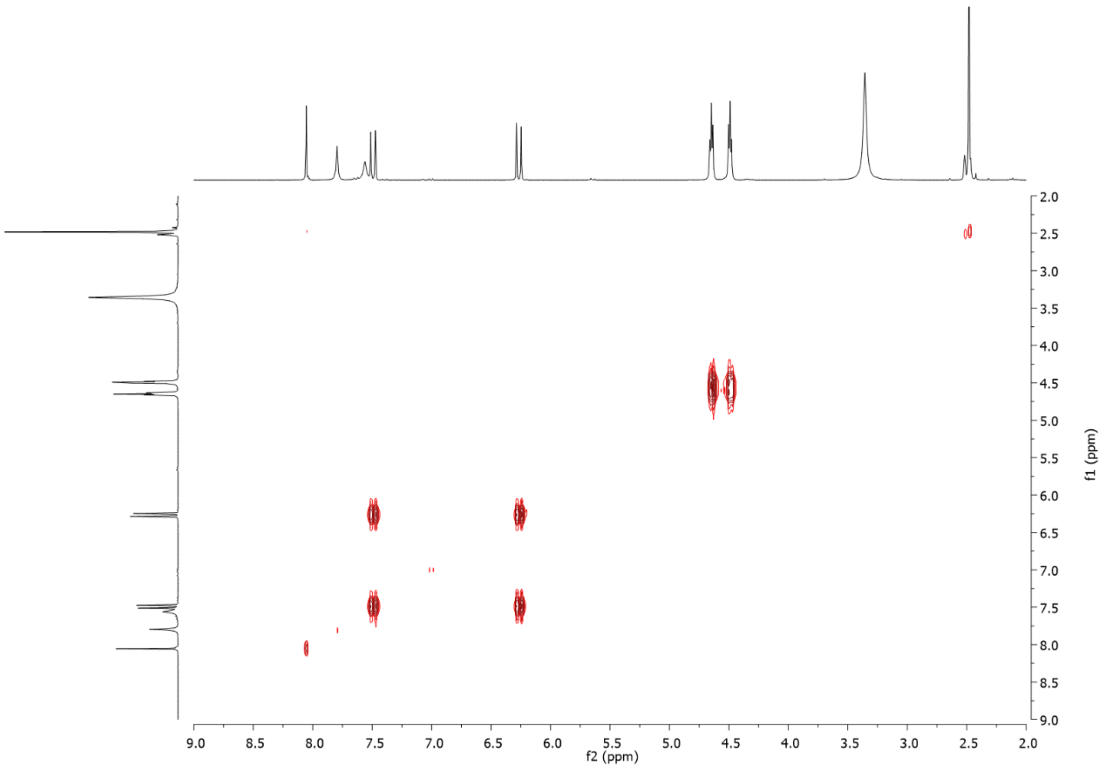

Figure S7. COSY spectrum of MTZH-2 in $(CD_3)_2SO$.

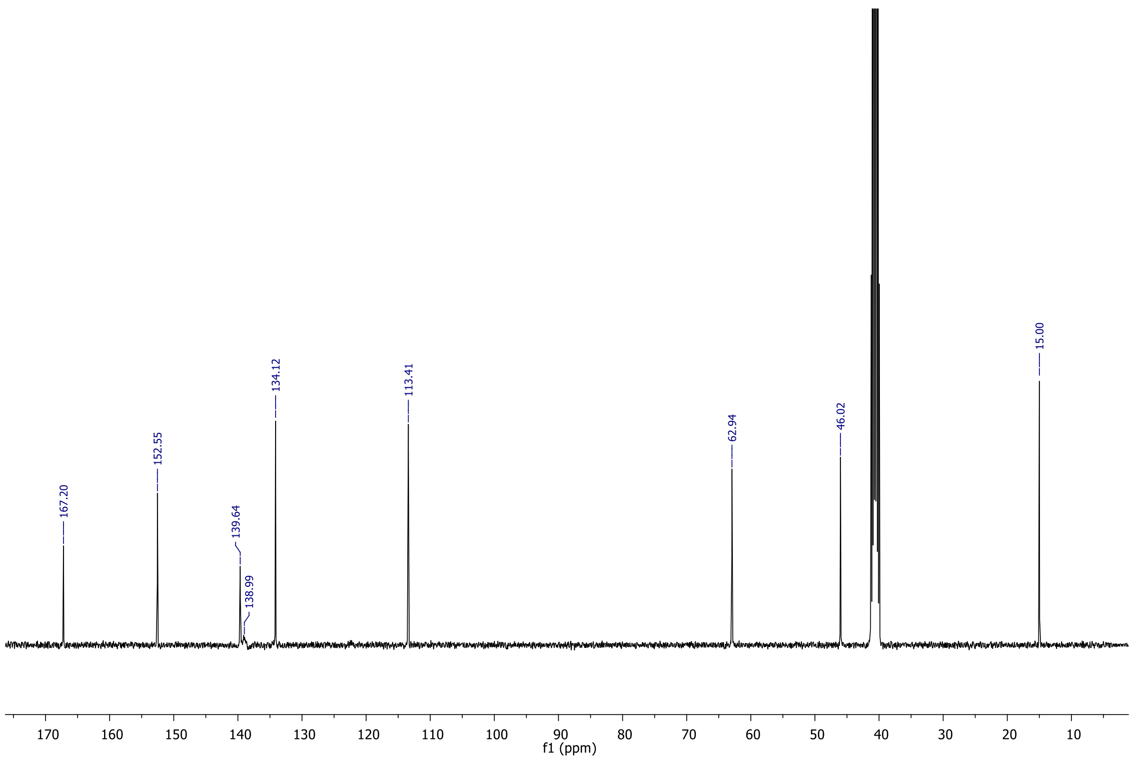

Figure S8. ^{13}C NMR (100 MHz) spectrum of MTZH-2 in $(CD_3)_2SO$.

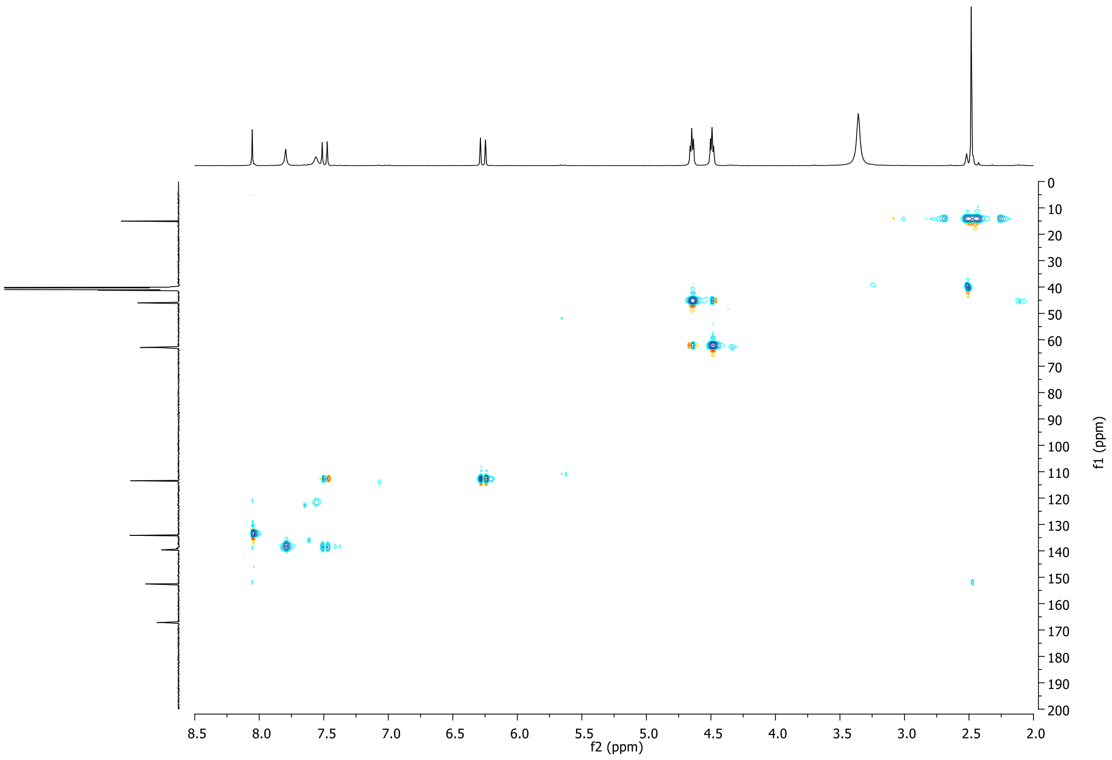

Figure S9. HSQC spectrum of MTZH-2 in $(CD_3)_2SO$.

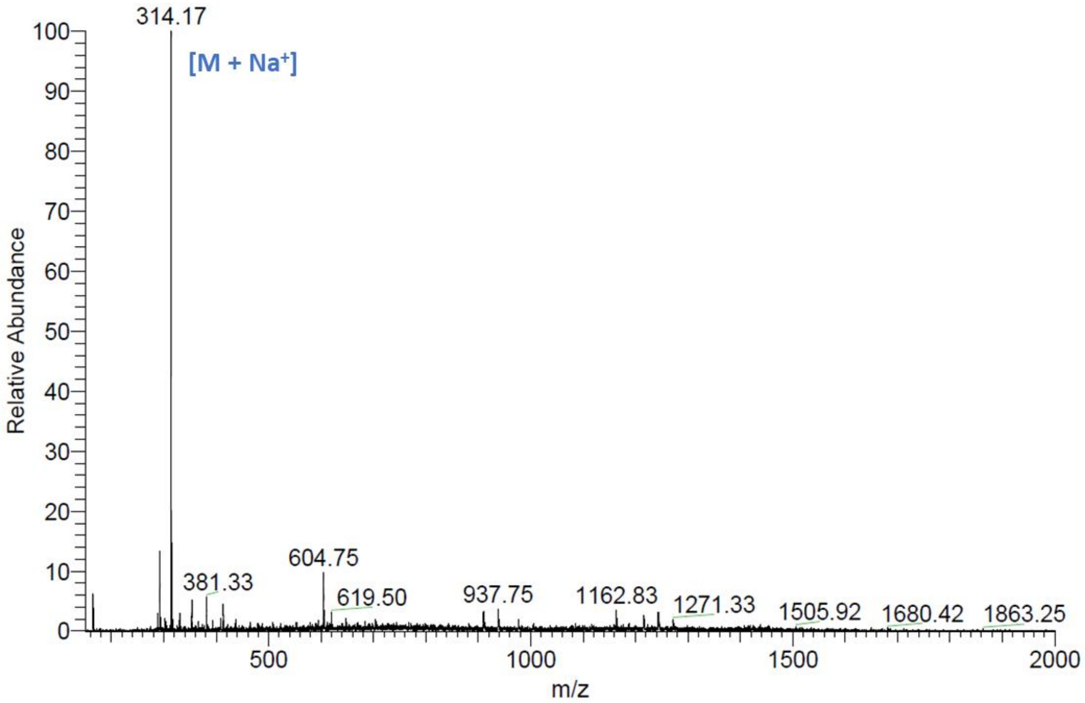

Figure S10. (ESI+)-MS spectrum of MTZH-2 (M) in methanol.

The ATR-FTIR analysis of MTZ-derivatives allows to easily detect the functional groups of the two ligands on the basis of diagnostic absorption bands. Indeed, as shown in Fig. S11 (SI), both the IR spectra of MTZH-1 and MTZH-2 display the typical absorption bands of the nitroimidazole moiety, such as the sharp ones centered at around 1533, 1366 and 1184 cm^{-1}, that can be attributed to the stretching vibration of NO_2/NO, N=O and of a tertiary amine group of this fragment.[1] The stretching of the carbonyl function of the two ligands is lowered by *ca.* 20 cm^{-1} in MTZH-2 compared to MTZH-1 (ν = 1703.14 *vs* 1724.36 cm^{-1}), accordingly with the lower frequencies usually reported for α,β-unsaturated esters relative to ester groups[2] and, in case of MTZH-2, the sharp band at 1635.64 cm^{-1} can be taken as diagnostic of the C=C stretching of the vinyl acetate group.[3]

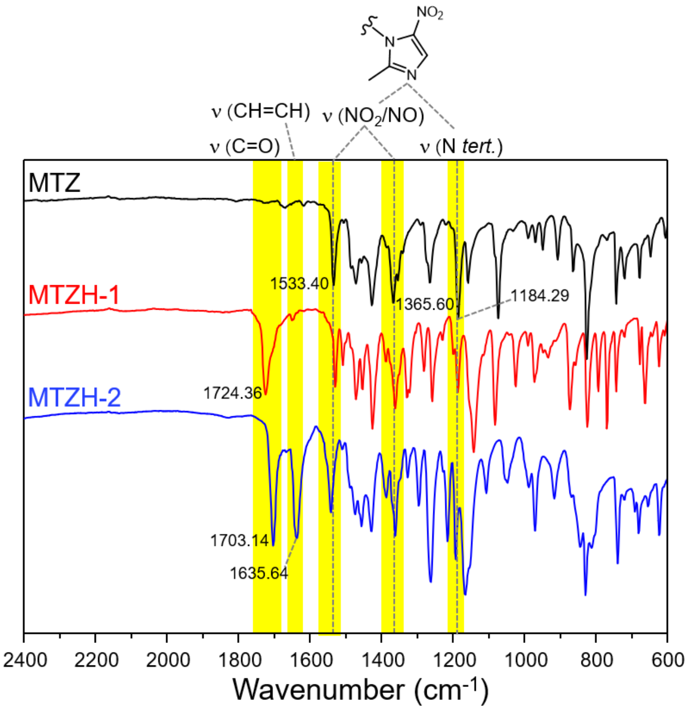

Figure S11. ATR-FTIR spectra of MTZH-1 (red) and MTZH-2 (blue), compared to the one of MTZ (black) in the 2400 - 600 cm^{-1} range of wavenumbers.

1.2. X-Ray Crystallography

The structure of MTZH-2 ligand was characterized by X-ray single crystal analysis. Crystals of MTZH-2 were obtained by slow evaporation of a concentrated solution (5 mM) of the ligand in H_2O/MeOH (30:70, v/v).

X-ray data collection was performed on a Bruker D8 Venture equipped with IµS 3.0 Microfocus Incoatec Source and a Photon III Detector. The Cu K_α radiation was used for the two data collections, performed at a temperature of 100 K (CryoStream 700 by Oxford Cryosystems). The dataset was collected with the APEX II then it was reduced and refined with the program SAINT by Bruker (APEX3 v2018.7-0, Copyright 2003, 2004 Bruker Nonius; Copyright 2005-2018 Bruker AXS). Absorption correction was applied by means of the SADABS program[4] included in the Bruker Package of data treatment. To solve the structure of MTZH-2, the program Shelxt 2018/3 was used and the refinement was carried on with Shelxl[5] by full-matrix least-squares techniques with anisotropic displacement parameters for all non-hydrogen atoms. All the hydrogen atoms but those of the water molecules, and the one on N4B, were placed in calculated positions and refined considering a riding model with isotropic thermal parameters. The other hydrogen atoms were found in the electron density map and refined isotropically.

The crystal selected for the data collection was the best found in different batches of crystallization experiments. Notwithstanding the diffraction quality was not excellent, as denoted by quite high R values and θ diffraction below to 95.9°, the structure was unambiguously determined. A nice hydrogen bond network can be also identified even though the hydrogen atoms placement led to some warnings in the checkcif file.

CCDC Deposition Number 2231746 contains the supplementary crystallographic data for this paper. These data can be obtained free of charge from the Cambridge Crystallographic Data Centre (CCDC) via http://www.ccdc.cam.ac.uk/Community/Requestastructure.

Table S1. Crystal data and structure refinement parameters for MTZH-2.

	MTZH-2
Empirical formula	C_{26} H_{38} $N_{10}O_{12}$
Formula weight	682.66
Temperature (K)	100(2)
Wavelength (Å)	1.54178
Crystal system, space group	Triclinic, P-1
Unit cell dimensions (Å)	a = 7.0235(3) α = 81.576(3)<br>b = 13.1846(6) β = 88.158(3)<br>c = 16.8650(8) γ = 81.022(3)
Volume ($Å^3$)	1525.89 (12)
Z, D_c (mg/cm^3)	2, 1.486
μ (mm^{-1})	1.015
F(000)	8720
Crystal size (mm)	0.04 x 0.4 x 0.8
θ range (°)	2.65 < θ < 95.9
Reflections collected / unique	8333/4489
Data / restraints / parameters	4489/0/460
Goodness-of-fit on F^2	1.051
Final R indices [I>2σ(I)]	R1 = 0.0910 wR2 = 0.250
R indices (all data)	R1 = 0.118 wR2 = 0.280

Table S2. Selected dihedral angles for the two molecules (A and B) in the a.u.

N5A-C1A-N2A-C4A	-1.40 (0.49)	N5B-C1B-N2B-C4B	179.68 (0.40)
N5A-C1A-N2A-C6A	9.03 (0.71)	N5B-C1B-N2B-C6B	-5.90 (0.67)
N2A-C6A-C7A-O3A	-63.98 (0.43)	N2B-C6B-C7B-O3B	67.79 (0.41)
C5A-C4A-N2A-C6A	-3.43 (0.68)	C5B-C4B-N2B-C6B	6.11 (0.65)

As shown in Figure S12a, the asymmetric unit (*a.u.*) of MTZH-2 contains two molecules of the ligand (herein referred as A and B), held in place by the hydrogen bond between the HN4B atom of the imidazole moiety of a B molecule and the acceptor nitrogen atom N1A gathered on the nitroimidazole fragment of the A unit (H--N distance 1.94(6) Å).

The two molecules crystalized with different orientation (see Table S2 for the selected dihedral angles). It is also possible to highlight the occurrence of intramolecular interactions involving the NO_2 group of the nitroimidazole moiety and the nearest aliphatic H atom, that impart a bended conformation to ligands. In the *a.u.* are also present different solvent molecules (two methanol and two water molecules), which form interesting networks of hydrogen bonds, both intramolecular and intermolecular. Looking down along *a*, a channel through the whole crystal can be identified, the wall of which is defined

by the imidazole/nitroimidazole moieties of ligands kept together by intermolecular "nitrogen" hydrogen bond and by stacking interactions involving the imidazole/nitroimidazole rings gathered on two different molecules, one of the original set and the other reported by the symmetry operation x-1, y, z (Figure S12b). In the channel resulting from such disposition are located all the solvent molecules identified in the crystal.

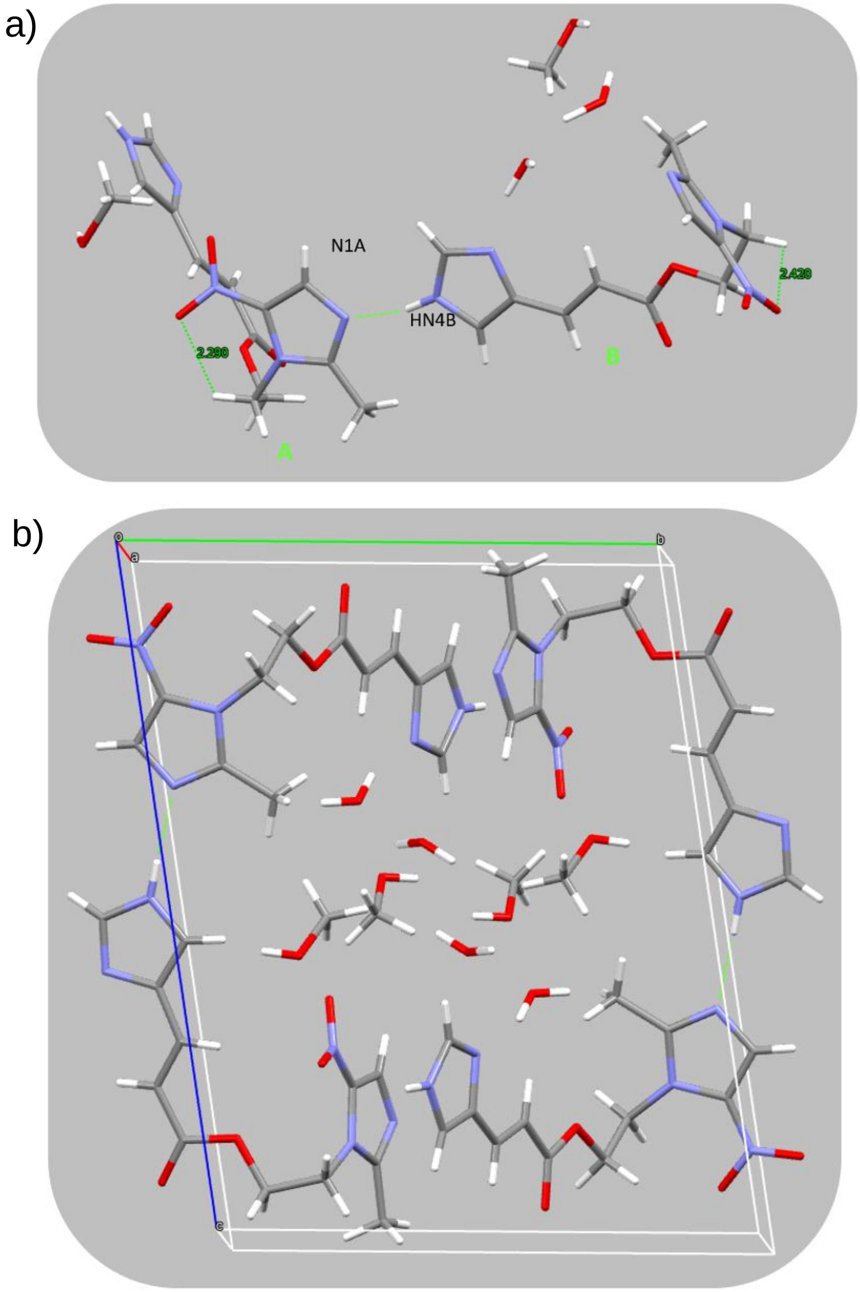

Figure S12. Asymmetric unit (*a.u.*) of MTZH-2 containing two molecules of the ligand plus two molecules of methanol and water (a) and *hole*-*wall* disposition of the crystal (b) evidencing the *wall*, defined by the molecules of ligand, and the channel through the crystal (*hole*), where the solvent molecules are located.

Table S3. Atomic charges on nitrogen atoms of MTZH-1 and MTZH-2, as determined by means of DFT calculations.

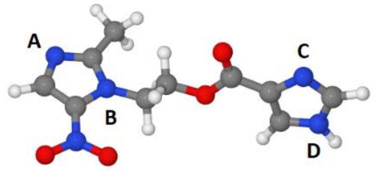

	Mulliken	Lowdin	Natural
A	-0.240	-0.240	-0.487
B	-0.082	-0.185	-0.395
C	-0.285	-0.202	-0.440
D	-0.512	-0.320	-0.573

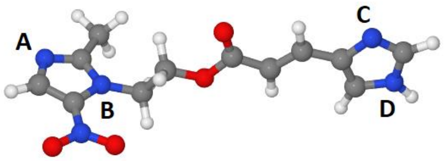

	Mulliken	Lowdin	Natural
A	-0.242	-0.241	-0.487
B	-0.095	-0.185	-0.394
C	-0.314	-0.241	-0.467
D	-0.482	-0.317	-0.569

1.3. Characterization of ruthenium complexes

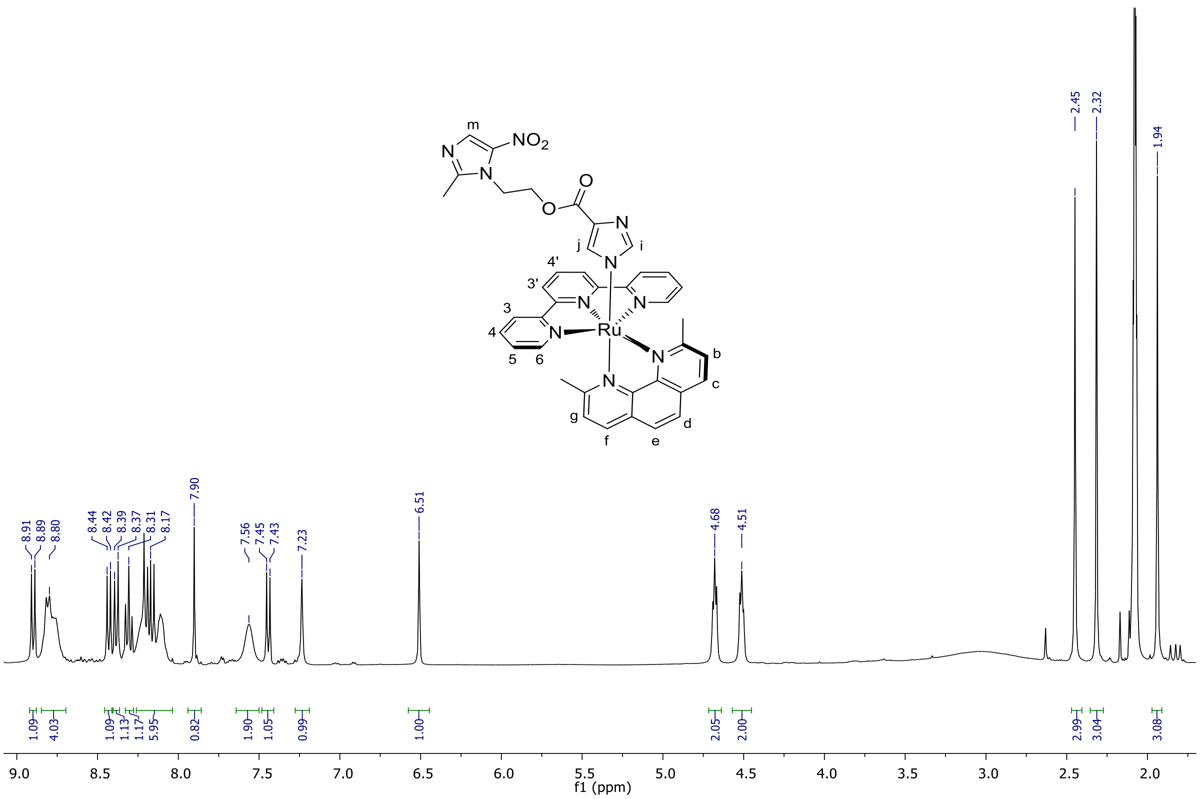

Figure S13. ^{1}H NMR (400 MHz) spectrum of **Ru2** in $(CD_3)_2CO$.

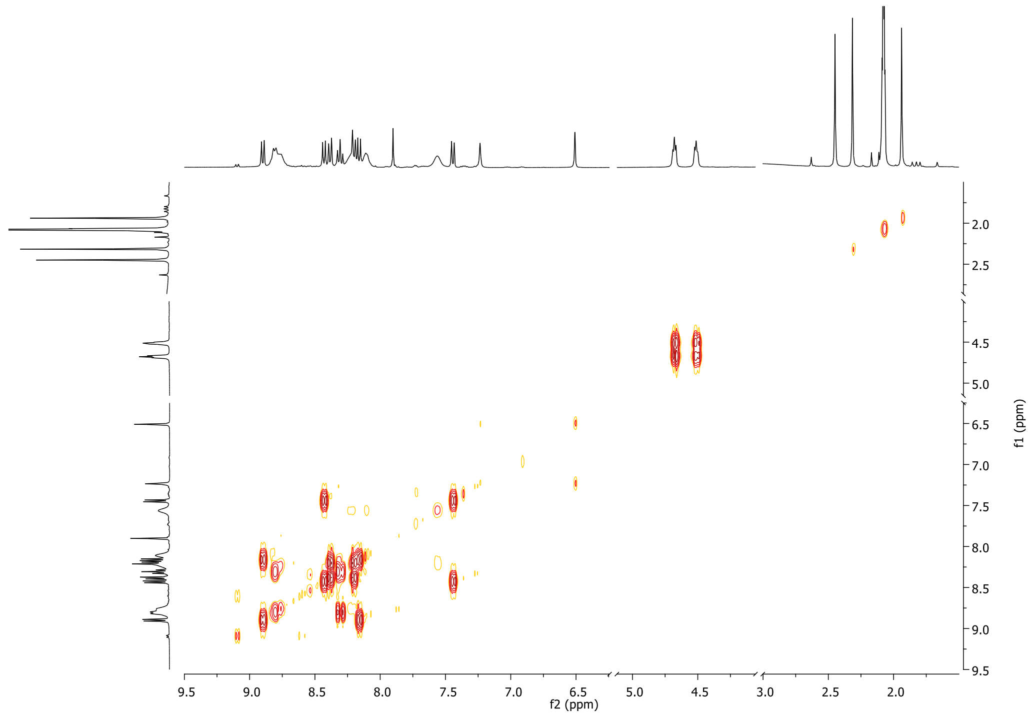

Figure S14. COSY spectrum of **Ru2** in $(CD_3)_2CO$.

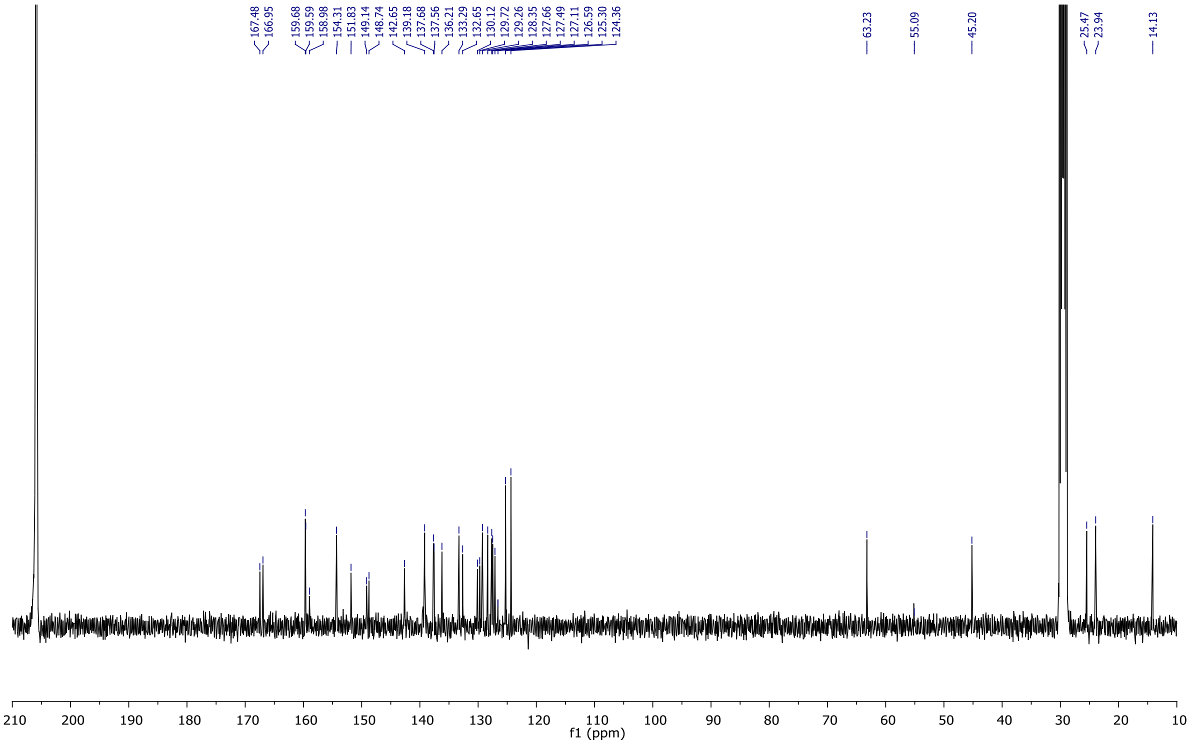

Figure S15. ^{13}C NMR (100 MHz) spectrum of **Ru2** in $(CD_3)_2CO$.

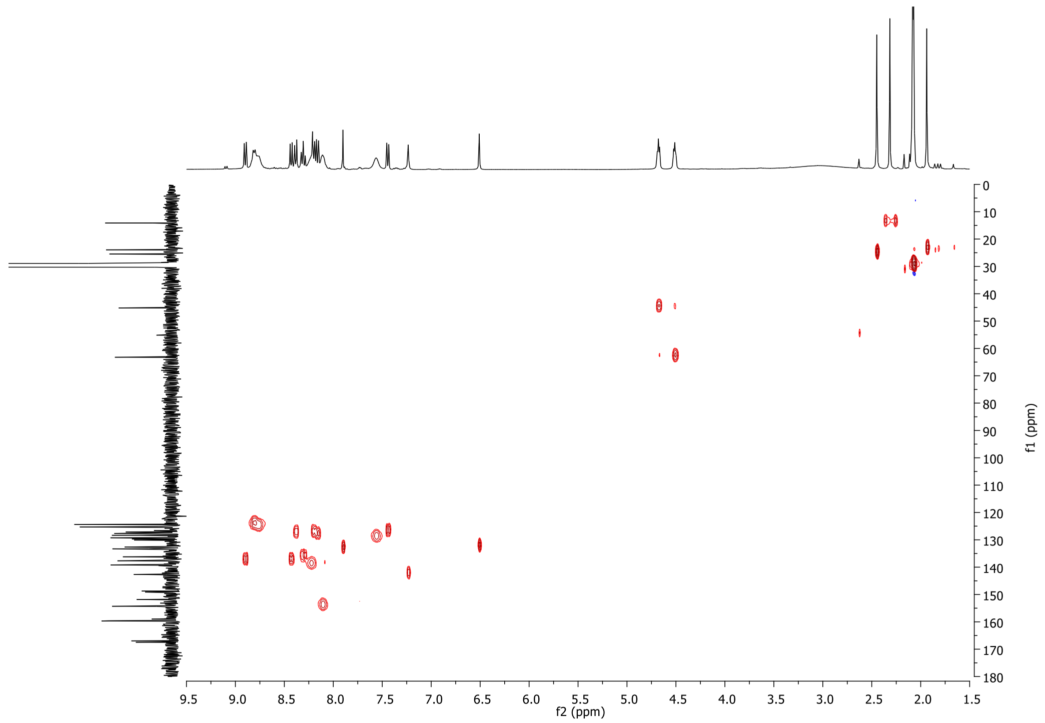

Figure S16. HSQC spectrum of **Ru2** in $(CD_3)_2CO$.

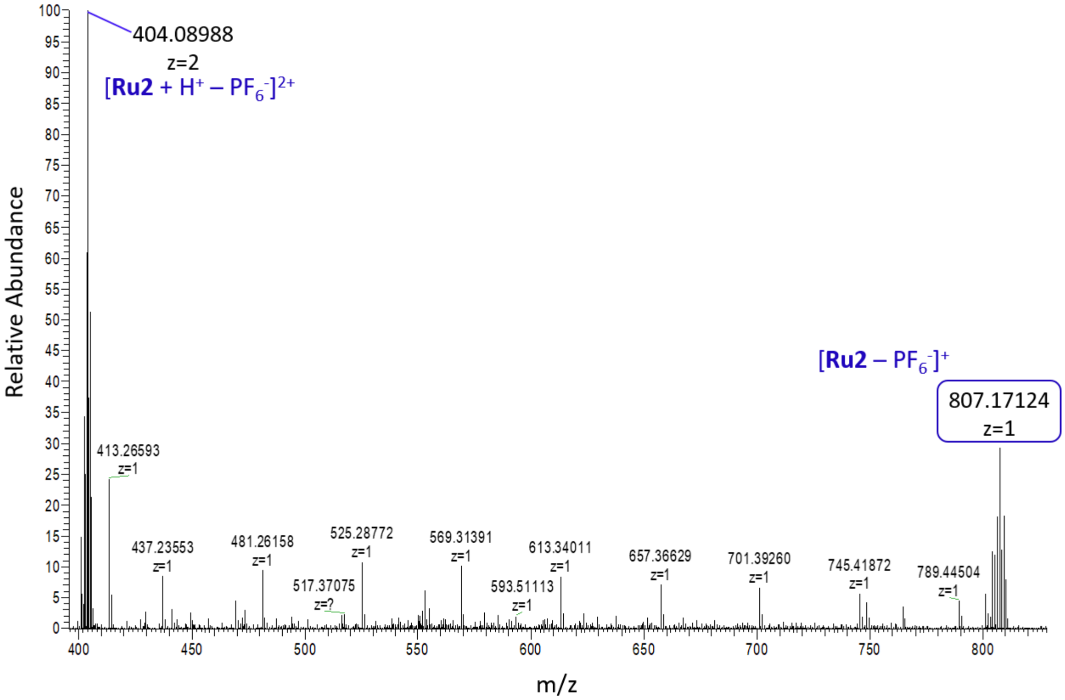

Figure S17. HR-MS (ESI+) spectrum of ruthenium complex **Ru2** in acetonitrile.

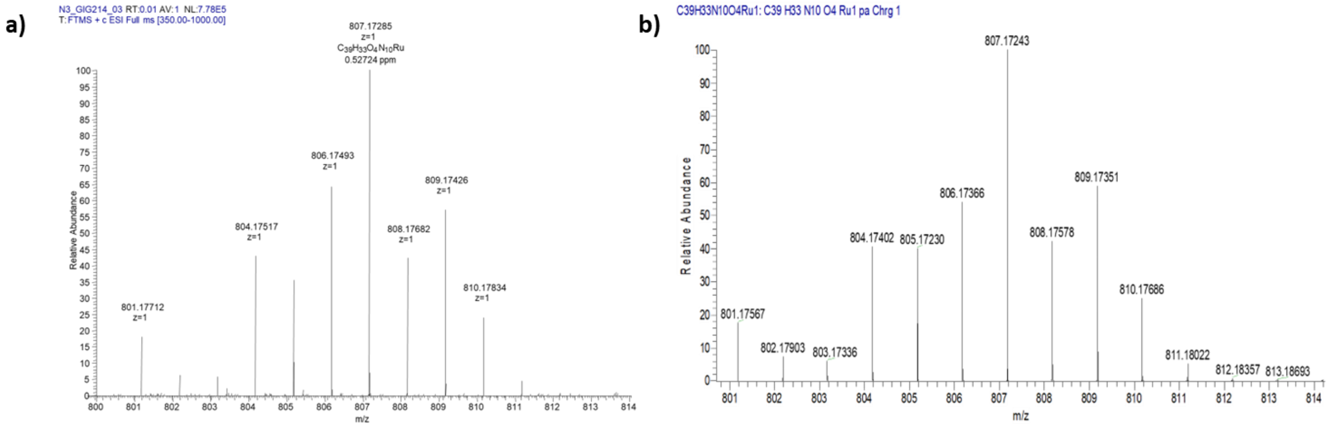

Figure S18. Isotopic pattern of the [R**u2** - PF_6^-]$^+$ (z = 1) ion, with measured (a) and theoretical (b) m/z values.

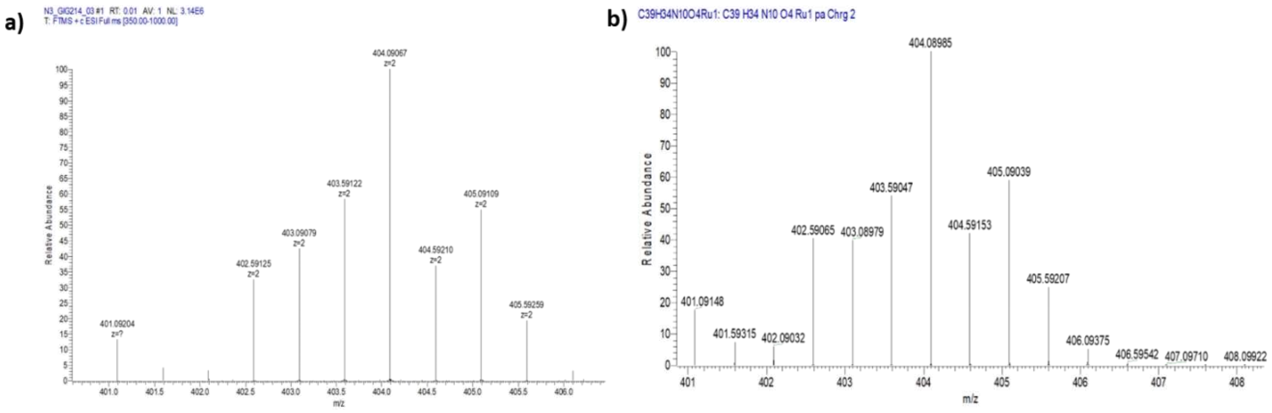

Figure S19. Isotopic pattern of the [**Ru2** + H^+- PF_6^-]$^{2+}$ (z = 2) ion, with measured (a) and theoretical (b) m/z values.

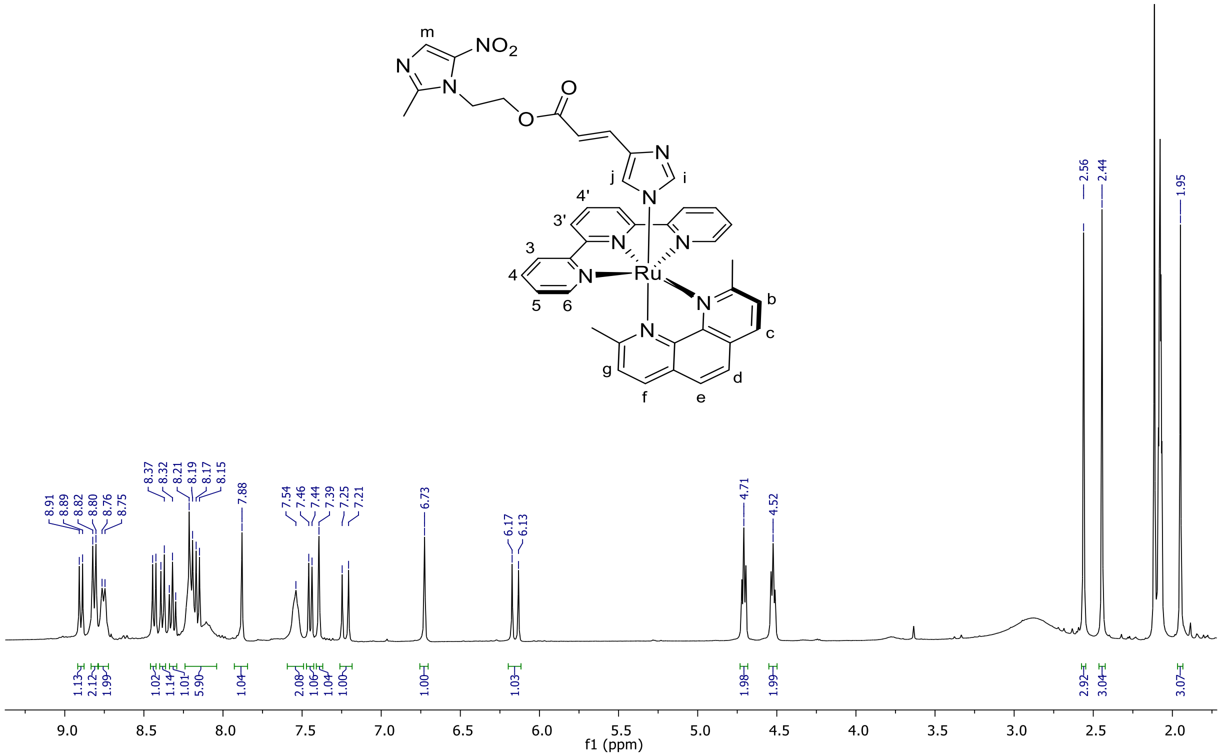

Figure S20. ^{1}H NMR (400 MHz) spectrum of **Ru3** in $(CD_3)_2CO$.

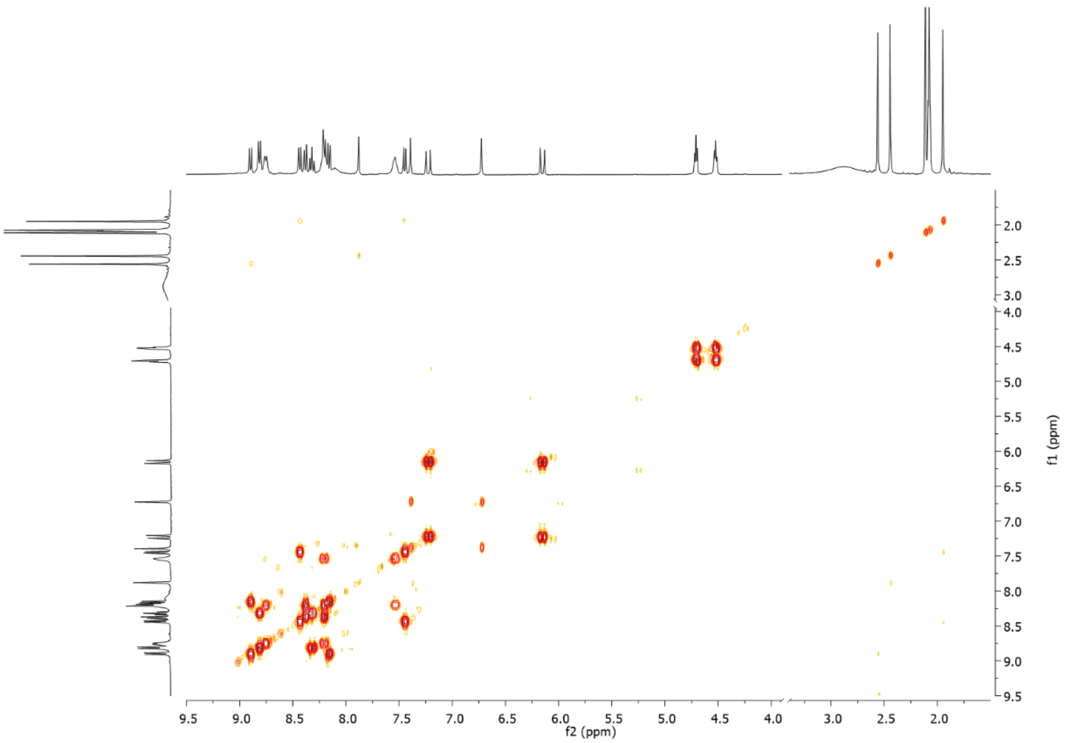

Figure S21. COSY spectrum of **Ru3** in $(CD_3)_2CO$.

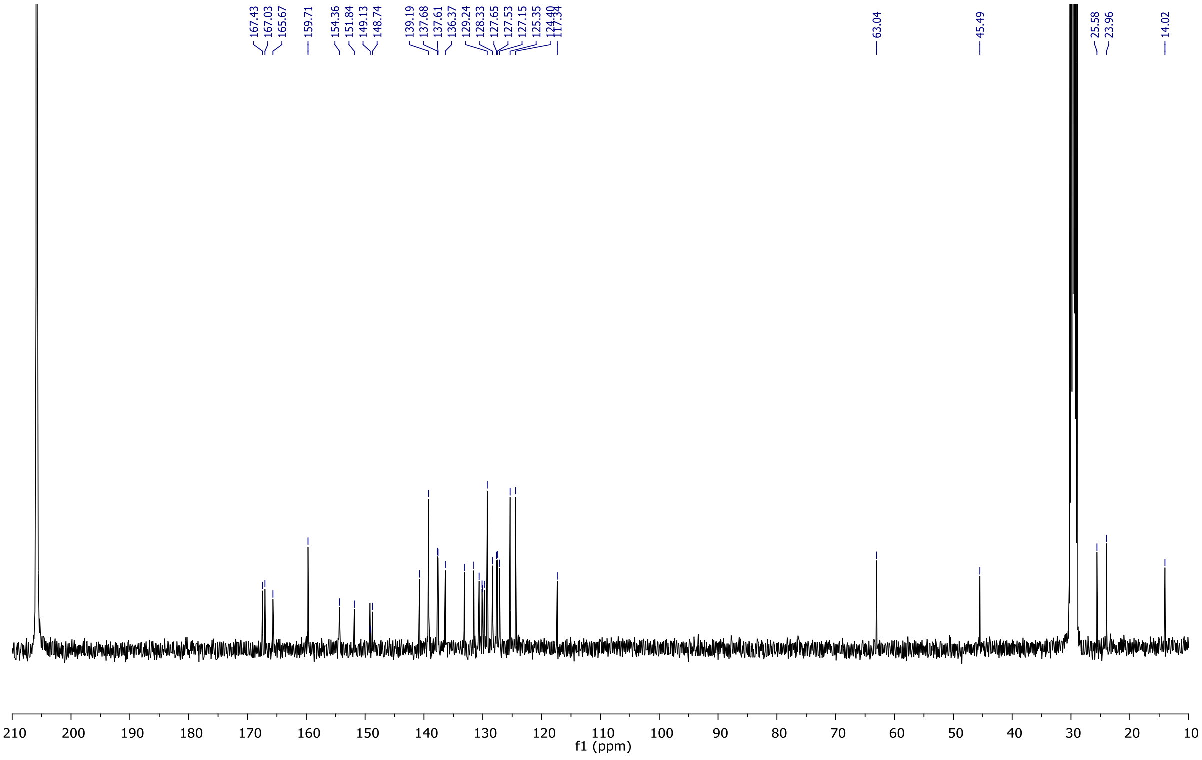

Figure S22. ^{13}C NMR spectrum of **Ru2** in $(CD_3)_2CO$.

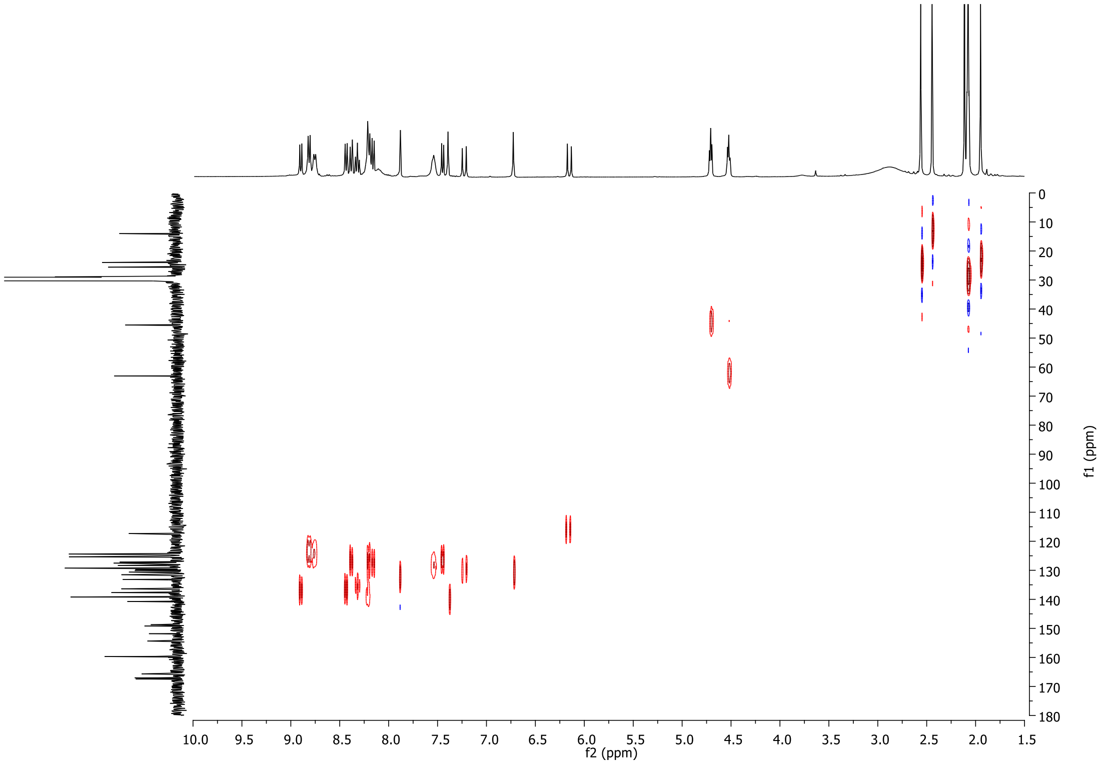

Figure S23. HSQC spectrum of **Ru3** in $(CD_3)_2CO$.

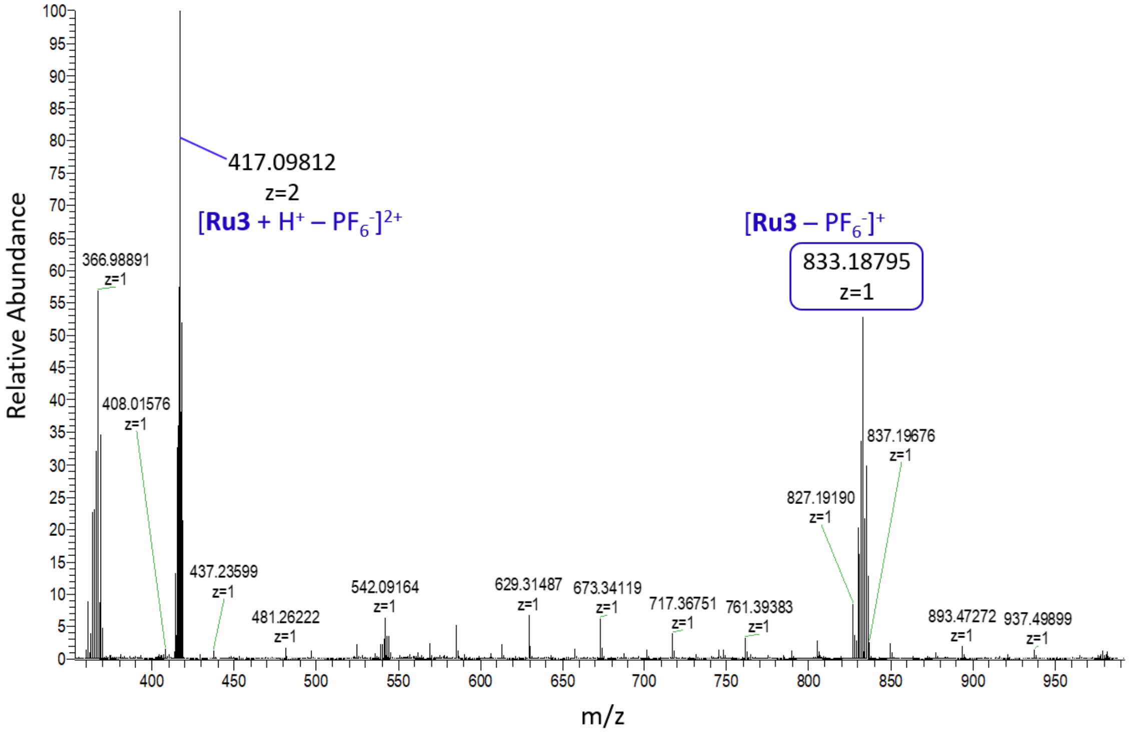

Figure S24. HR-MS (ESI+) spectrum of ruthenium complex **Ru3** in acetonitrile.

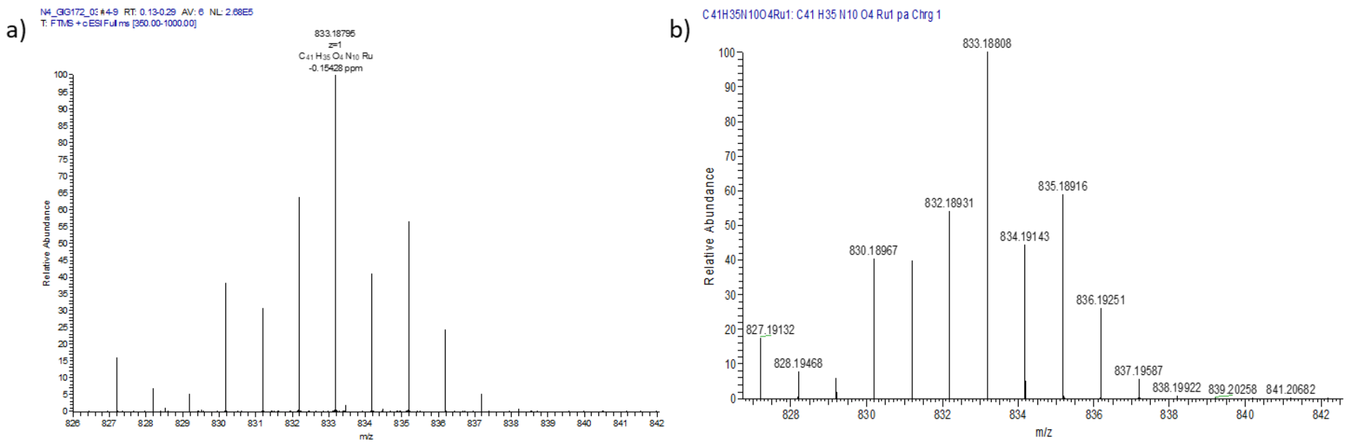

Figure S25. Isotopic pattern of the [**Ru3** - PF_6^-]$^+$ (z = 1) ion, with measured (a) and theoretical (b) m/z values.

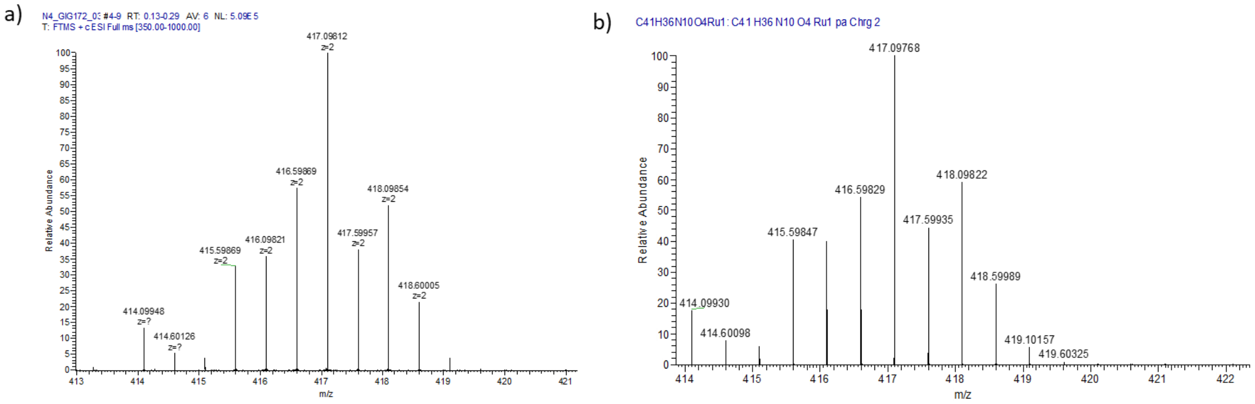

Figure S26. Isotopic pattern of the [**Ru3** + H^+- PF_6^-]$^{2+}$ (z = 2) ion, with measured (a) and theoretical (b) m/z values.

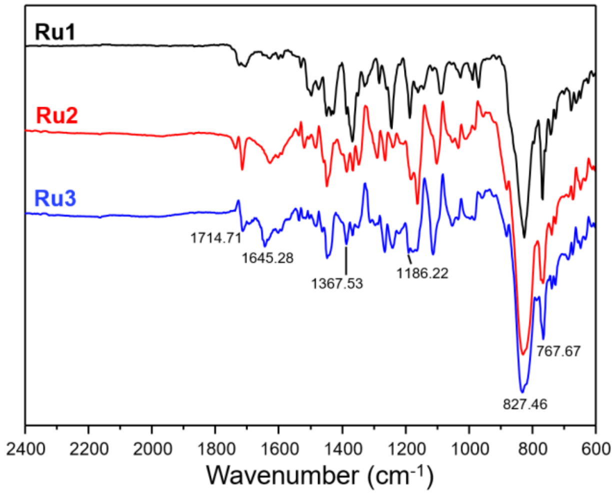

Figure S27. ATR-FTIR spectra of ruthenium complexes **Ru1**, **Ru2** and **Ru3**. In the fingerprint region the strong absorption at 827.46 cm^{-1} is due to the hexafluorophosphate counter-ion present in the molecules.[6]

2. Photoreactivity of ruthenium complexes

2.1. Stability of ruthenium compounds under dark

Prior to evaluate the photoreactivity of **Ru2** and **Ru3**, we investigated their stability under dark in acetonitrile and in aqueous solutions. This was performed by monitoring the absorption spectra of solutions at fixed concentration of the two Ru(II) compounds with increasing incubation times under dark: the obtained results in acetonitrile and in water are respectively shown in Figures S28 and S29.

As shown, both the absorption profiles of **Ru2** and **Ru3** did not exhibit appreciable variations over a total period of 60 h *in dark*, thus unveiling a remarkable stability of metal complexes under these conditions. An analogues behavior was also previously observed for **Ru1**.

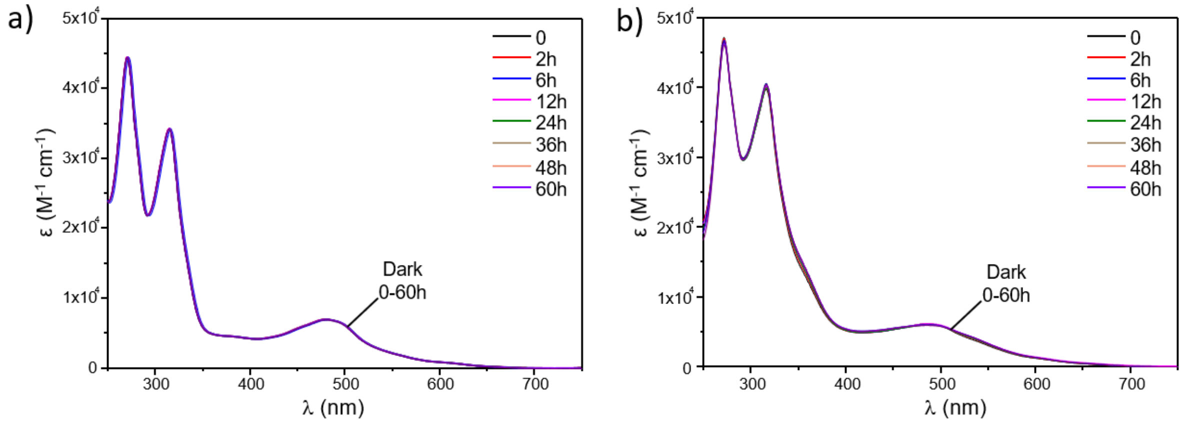

Figure S28: Electronic absorption spectra of **Ru2** (a) and **Ru3** (b) in acetonitrile collected with increasing incubation times under dark, over a total period of 60 h.

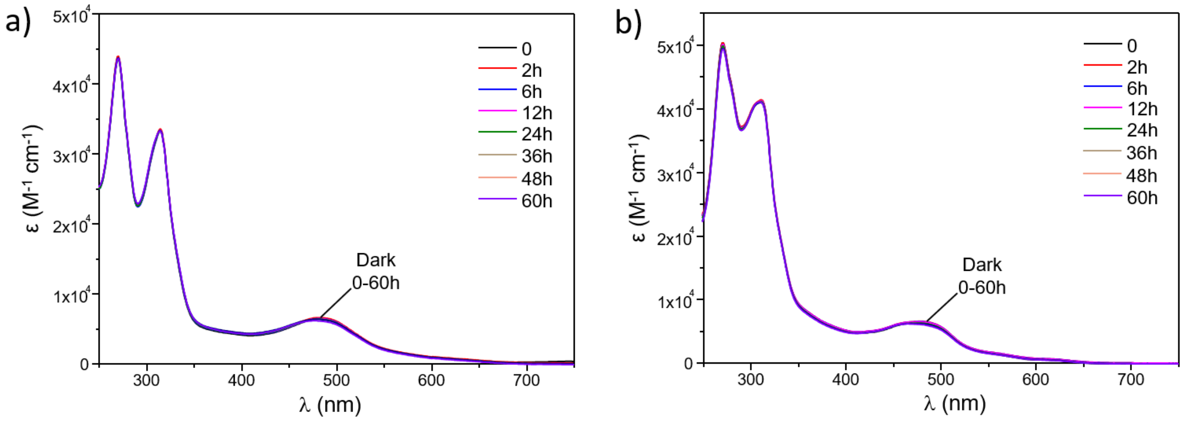

Figure S29: Electronic absorption spectra of **Ru2** (a) and **Ru3** (b) in aqueous solutions (PBS, pH 7.4) collected with increasing incubation times under dark at 37 °C, over a total period of 60 h.

2.2. Photoejection of MTZ derivatives

The ability of **Ru2** and **Ru3** to effectively undergo ligand photosubstitution was investigated in acetonitrile and in aqueous media (PBS buffer, pH 7.4), and monitored through UV-Vis and HPLC analysis. The obtained results for **Ru2** and **Ru3** are respectively reported in Figure 2 of the manuscript and in Figures S30-31.

Chromatographic conditions for the HPLC analysis were optimized by using as eluent a gradient mixture of H_2O/CH_3CN acidified with 0.1% of formic acid, as specified below.

The quantum yields (Φ_{434} values) for the photodissociation of MTZ derivatives from **Ru2** and **Ru3** in acetonitrile and water were respectively determined through UV-Vis spectroscopy and HPLC analysis, following the previous determination of the photon flux of the light source performed as already described.[7] The resulting Φ_{434} values are compared together with those previously determined for **Ru1** in Table 1 of the main text.

Table S4. HPLC gradient used for photoejection studies.

time (min)	% H_2O (0.1% HCOOH)	% CH_3CN (0.1% HCOOH)
0	98	2
2	95	5
5	85	15
10	70	30
15	40	60
20	5	95
25	98	2

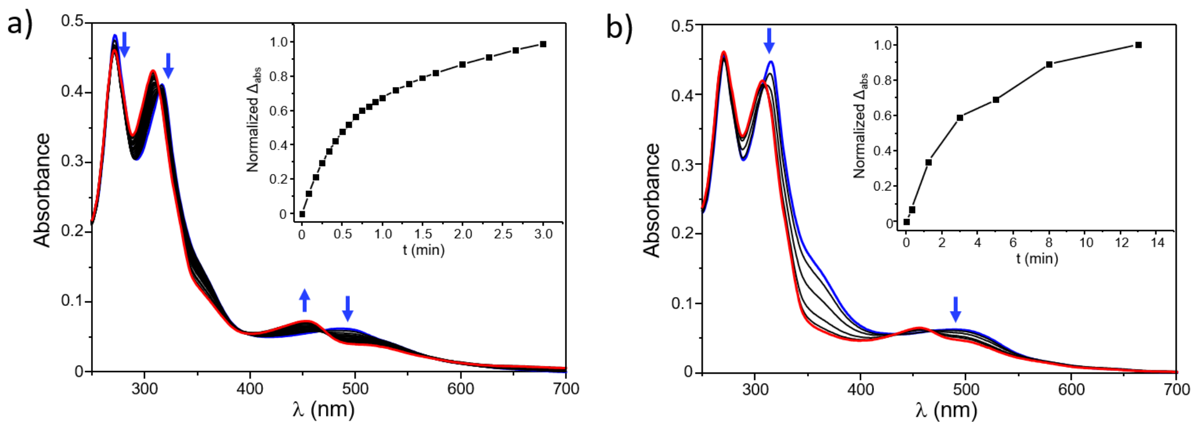

Figure S30. Electronic absorption spectra of **Ru3** in acetonitrile (a) and in aqueous solution (b) subjected to increasing irradiation times.

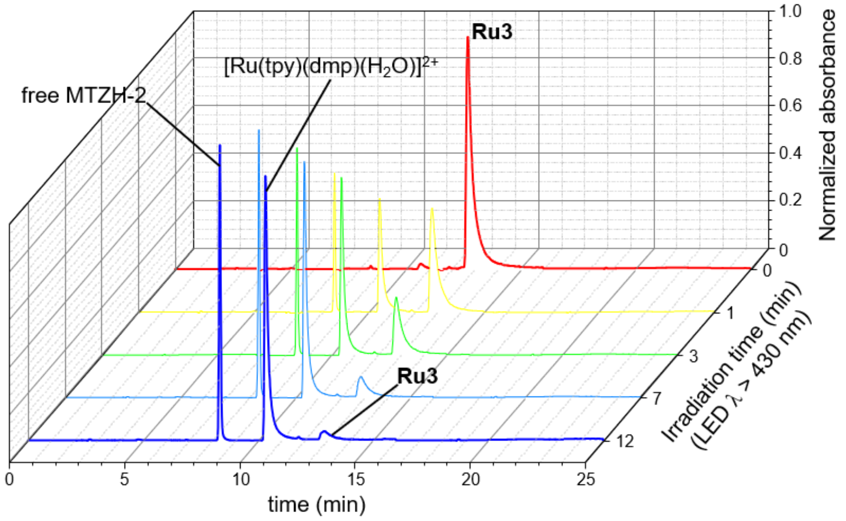

Figure S31: HPLC chromatograms of **Ru3** in aqueous solution subjected to progressive irradiation times.

2.3. Singlet oxygen generation

The ability of ruthenium compounds to sensitize the formation of oxygen ($^1O_2$) upon light-activation was investigated through UV-Vis spectroscopy, by using 1,5-dihydroxynaphthalene (DHN), as indirect reporter of $^1O_2$. This method relies on the prompt and quantitative oxidation of DHN by $^1O_2$ to give 5-hydroxy-1,4-naphthalenedione (Juglone), according to Eq. S1 and Scheme S1:

$$\mathrm{DHN} + {}^1\mathrm{O}_2 \rightarrow \mathrm{Juglone} + \frac{1}{2}\,{}^3\mathrm{O}_2 \qquad (\mathrm{Eq.}\ S1)$$

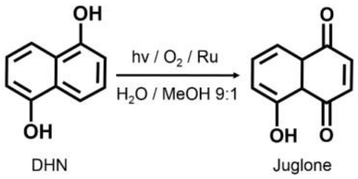

Scheme S1. Photochemical conversion of DHN to Juglone.

This process can be easily monitored by following the decrease of the DHN absorption band, centered at *ca.* 297 nm, and the simultaneous increase of the broad band of Juglone, which is centered at 427 nm.

The experiments were performed according to literature and described in the experimental section of the main text. The resulting UV-Vis analysis recorded for **Ru2** and **Ru3** are respectively shown in Fig. S32a and S32b.

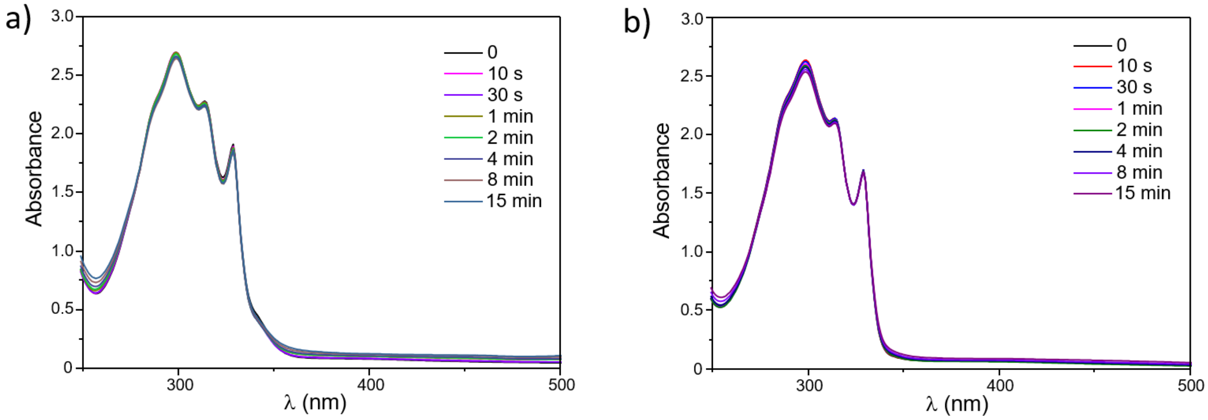

Figure S32. Analysis of the singlet oxygen sensitizing properties of **Ru2** (a) and **Ru3** (b) performed spectrophotometrically by using DHN as indirect $^1O_2$ reporter. In figures are shown the absorption spectra of aqueous solutions of ruthenium compounds ([Ru] = 10 µM, pH 7.4) containing DHN ([DHN] = 3.3×10^{-4} M) and subjected to progressive irradiation times for a total period of 15 min. Spectra were acquired by using as blank reference a solution containing only the Ru-compound.

3. Cellular internalization of ruthenium complexes

The ability of ruthenium complexes to be internalized into bacterial cells was inspected by means of Inductively Coupled Plasma Atomic Emission Spectroscopy (ICP-AES), by adopting an experimental procedure previously described.[8] Briefly, *B. subtilis* bacterial cultures, chosen as a model of Gram-positive bacteria, were added to 1.5 mL polyproprilene tubes (7.5 × 10^8 cells/tube) and incubated at 37 °C for 30 min with a fixed concentration of ruthenium compounds (200 µM). Then, bacterial cells were centrifuged, and cellular pellets were washed three times with 1 mL 0.9% NaCl solution to remove extracellular ruthenium compounds present in the medium. In the washed bacterial cell pellets the ruthenium-content was assumed as indicator for drug permeation and was determined by ICP-AES, by using the 267.876 and 245.657 nm wavelengths. The experiments were performed in triplicate and the results, expressed as ng of Ru/10^8 cells, are shown in Figure S33.

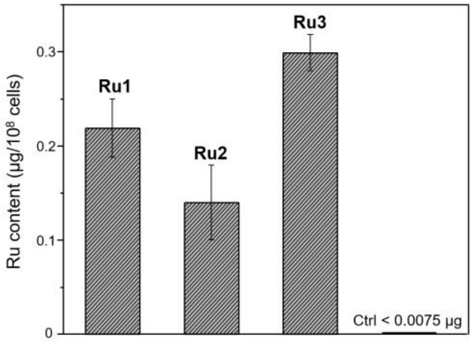

Figure S33. Cellular uptake of ruthenium compounds **Ru1**, **Ru2** and **Ru3** after 30 min incubation with *B. subtilis* obtained by means of ICP-AES analysis.

4. Determination of inhibitory concentrations

The antibacterial activity of nitroimidazole-based compounds and ruthenium complexes were investigated on *B. subtilis* strain 168. Experiments were performed in dark and following blue light irradiation (LED emitting at 434 nm, t = 40 min), both in aerobiotic and in anaerobiotic conditions, as described in the experimental section of the main text. Growth analysis were done on microtiter plates incubated on an Infinite Pro 200 plate reader (Tecan, Switzerland) at 37 °C. Obtained values of OD_{600} nm are reported in Figure 4 of the manuscript whereas the differences in the growth were statistically evaluated by one-way ANOVA test and Tukey post-hoc test (Table S5).

For the experiments it was employed a specifically engineered anaerobiosis jar, illustrated in Figure S34. As shown, the jar contained a tray for the multiwell plate held at fixed distance (5 cm) from the LED source, switchable through an external magnetic light switch without perturbing the internal environment of the jar. Anaerobic conditions were obtained by introducing Thermo Scientific AnaeroGen™ into the 2.5 l jar and checking the obtained anerobic conditions by using the Thermo Scientific™ Resazurin indicator.

The photon flux in each plate by using this experimental set up was calculated to be 4.72×10^{-9} E/s, as determined by the ferrioxalate actinometer method.[9]

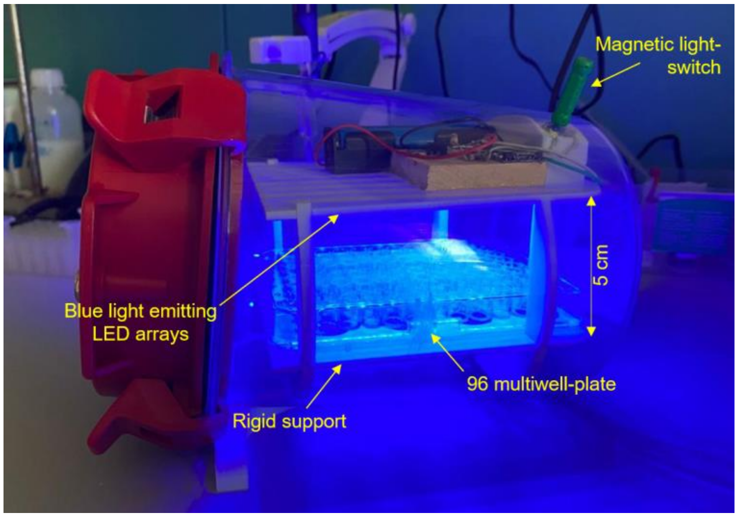

Figure S34: Experimental set up employed in the antibacterial activity tests reported in this work.

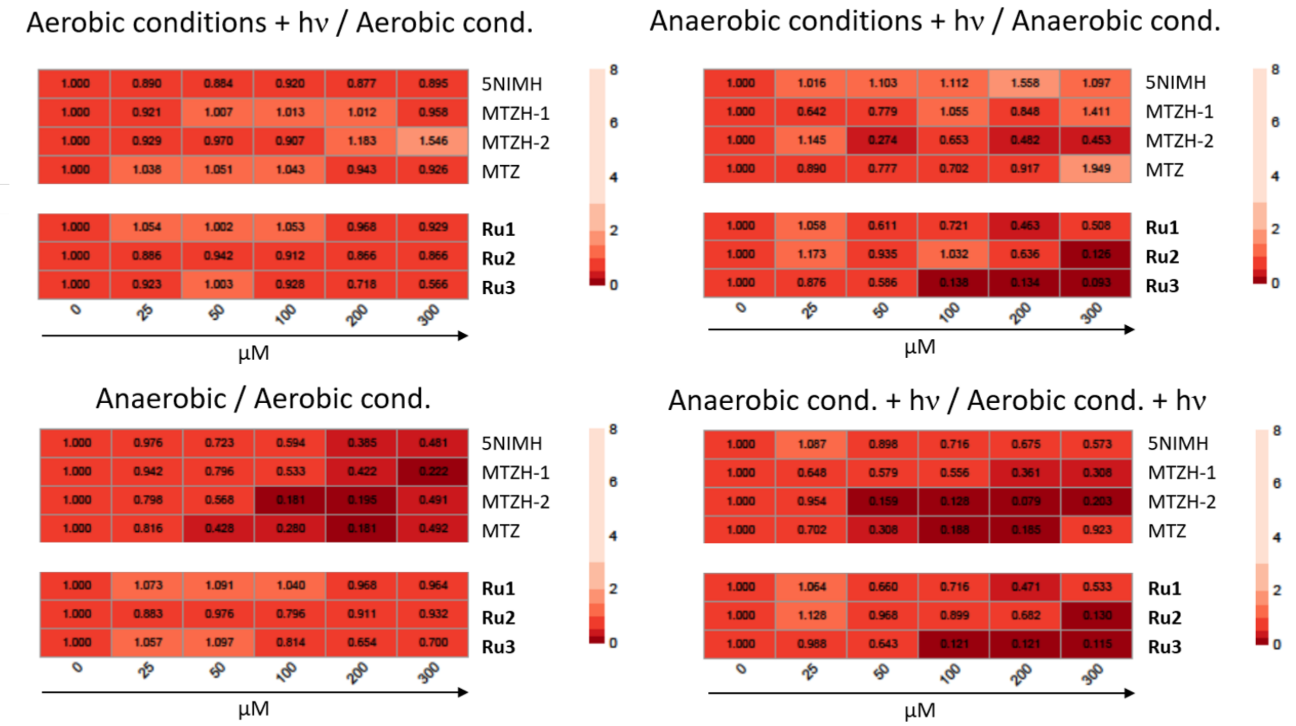

Figure S35: Pairwise comparison between the antibacterial effect observed in different conditions. Lower values (dark color) indicate lower growth in the first condition tested compared to the second one.

	Dark		hν	
Ru conc.	50 µM	200 µM	50 µM	200 µM
**Ru1**	3.04 ± 0.02 x 10^7	3.04 ± 0.02 x 10^7	1.20 ± 0.03 x 10^7	5.00 ± 0.52 x 10^5
**Ru2**	2.50 ± 0.04 x 10^7	2.50 ± 0.04 x 10^7	1.58 ± 0.01 x 10^7	0
**Ru3**	3.39 ± 0.01 x 10^7	3.39 ± 0.01 x 10^7	1.00 ± 0.01 x 10^7	0

Table S5. Cell viability expressed as bacterial titres (Colony Forming Units, CFU/ml) after treatment of *B. subtilis* cell cultures with metal complexes in anaerobic conditions. Data are average and standard deviation from 4 independent experiments. Untreated controls titres were 3.74 ± 0.03 x 10^7 CFU/mL and 2.10 ± 0.02 x 10^7 CFU/mL for dark and light conditions, respectively.

REFERENCES

(1) Bensouiki, S.; Belaib, F.; Sindt, M.; Rup-Jacques, S.; Magri, P.; Ikhlef, A.; Meniai, A. H. Synthesis of Cyclodextrins-Metronidazole Inclusion Complexes and Incorporation of Metronidazole - 2-Hydroxypropyl-β-Cyclodextrin Inclusion Complex in Chitosan Nanoparticles. *J. Mol. Struct.* **2022**. https://doi.org/10.1016/j.molstruc.2021.131298.

(2) Orsini, F.; Ricca, G. S. Oxygen-17 NMR Chemical Shifts of Esters. *Org. Magn. Reson.* **1984**, *22* (10), 653–657. https://doi.org/10.1002/mrc.1270221011.

(3) Mixer, R. Y.; Heck, R. F.; Winstein, S.; Young, W. G. Cis- and Trans-Propenylbenzene and Their Azeotropes with n-Decane. *J. Am. Chem. Soc.* **1953**, *75* (16), 4094–4096. https://doi.org/10.1021/ja01112a521.

(4) Krause, L.; Herbst-Irmer, R.; Sheldrick, G. M.; Stalke, D. Comparison of Silver and Molybdenum Microfocus X-Ray Sources for Single-Crystal Structure Determination. *J. Appl. Crystallogr.* **2015**. https://doi.org/10.1107/S1600576714022985.

(5) Sheldrick, G. M. Crystal Structure Refinement with SHELXL. *Acta Crystallogr. Sect. C Struct. Chem.* **2015**. https://doi.org/10.1107/S2053229614024218.

(6) Adeloye, A. O.; Olomola, T. O.; Adebayo, A. I.; Ajibade, P. A. A High Molar Extinction Coefficient Bisterpyridyl Homoleptic Ru(II) Complex with Trans-2- Ethyl-2-Butenoic Acid Functionality: Potential Dye for Dye-Sensitized Solar Cells. *Int. J. Mol. Sci.* **2012**. https://doi.org/10.3390/ijms13033511.

(7) Giacomazzo, G. E.; Conti, L.; Guerri, A.; Pagliai, M.; Fagorzi, C.; Sfragano, P. S.; Palchetti, I.; Pietraperzia, G.; Mengoni, A.; Valtancoli, B.; Giorgi, C. Nitroimidazole-Based Ruthenium(II) Complexes: Playing with Structural Parameters to Design Photostable and Light-Responsive Antibacterial Agents. *Inorg. Chem.* **2022**, *61* (18), 6689–6694. https://doi.org/10.1021/acs.inorgchem.1c03032.

(8) Conti, L.; Mengoni, A.; Giacomazzo, G. E.; Mari, L.; Perfetti, M.; Fagorzi, C.; Sorace, L.; Valtancoli, B.; Giorgi, C. Exploring the Potential of Highly Charged Ru(II)- and Heteronuclear Ru(II)/Cu(II)-Polypyridyl Complexes as Antimicrobial Agents. *J. Inorg. Biochem.* **2021**, *220*, 111467. https://doi.org/10.1016/j.jinorgbio.2021.111467.

(9) Havrylyuk, D.; Deshpande, M.; Parkin, S.; Glazer, E. C. Ru(<scp>ii</Scp>) Complexes with Diazine Ligands: Electronic Modulation of the Coordinating Group Is Key to the Design of "Dual Action" Photoactivated Agents. *Chem. Commun.* **2018**, *54* (88), 12487–12490. https://doi.org/10.1039/C8CC05809A.
